# A comprehensive framework for computational modeling of growth and remodeling in tissue-engineered soft collagenous materials

**DOI:** 10.1007/s10237-025-01988-x

**Published:** 2025-07-23

**Authors:** M. Sesa, H. Holthusen, C. Böhm, S. Jockenhövel, S. Reese, K. Linka

**Affiliations:** 1 Institute of Applied Mechanics, RWTH Aachen University, Aachen, Germany; 2 Department of Biohybrid & Medical Textiles (BioTex), Institute of Applied Medical Engineering, RWTH Aachen University, Aachen, Germany; 3 University of Siegen, Siegen, Germany; 4 Institute for Continuum and Material Mechanics, Hamburg University of Technology, Hamburg, Germany

**Keywords:** Cardiovascular implants, Tissue engineering, Regenerative medicine, Anisotropic growth, Remodeling

## Abstract

Developing clinically viable tissue-engineered structural cardiovascular implants—such as vascular grafts and heart valves—remains a formidable challenge. Achieving reliable and durable outcomes requires a deeper understanding of the fundamental mechanisms driving tissue evolution during in vitro maturation. Although considerable progress has been made in modeling soft tissue growth and remodeling, studies focused on the early stages of tissue engineering remain limited. Here, we present a general, thermodynamically consistent model to predict tissue evolution and mechanical response throughout the in vitro maturation of passive, load-bearing soft collagenous constructs. The formulation utilizes a stress-driven homeostatic surface to capture volumetric growth, coupled with an energy-based approach to describe collagen densification via the strain energy of the fibers. We further employ a co-rotated intermediate configuration to ensure the model’s consistency and generality. The framework is demonstrated with two numerical examples: a uniaxially constrained tissue strip validated against experimental data and a cruciform-shaped biaxially constrained specimen subjected to load perturbation. These results highlight the potential of the proposed model to advance the design and optimization of tissue-engineered structural cardiovascular implants with clinically relevant performance.

## Introduction

Cardiovascular diseases (CVDs) are the primary cause of death worldwide (Di Cesare et al. 2024). Deaths attributed to CVDs have sharply increased in the last few decades (Amini et al. 2021). Tissue-engineered implants offer a promising long-term solution to treat CVDs by improving patients’ lifestyles compared to alternative implant types. This stems from their ability to grow, remodel, and adapt to hemodynamic conditions (Yacoub and Takkenberg 2005; Pashneh-Tala et al. 2016), combined with their resistance to calcification (Turner et al. 2024), making them a viable option to alleviate the limitations of existing cardiovascular implants. Tissue-engineered implants are fabricated in a process called the maturation process. Developing a maturation process capable of producing complex cardiovascular implants, such as heart valves or vascular grafts with sufficient mechanical properties remains a challenging task.

Cardiovascular tissue engineering encompasses different approaches, such as in vitro and in situ. In vitro tissue engineering involves culturing tissue constructs under controlled laboratory conditions before implantation, allowing precise control over the environment. In contrast, in situ tissue engineering relies on implanting biodegradable scaffolds directly into the body, where endogenous cells and biological signals drive tissue regeneration and remodeling at the site of implantation. Replicating conditions within the human body during in vitro experiments remains difficult, which often leads to discrepancies between the results of in vitro and in vivo experiments. An example is the concept of contact guidance, which is used to tailor the mechanical properties of textile-based tissue-engineered implants. Contact guidance was found to be ineffective in large animal experiments (Uiterwijk et al. 2020), contradicting findings from in vitro experiments (Foolen et al. 2012; Hermans et al. 2022). This underscores the necessity to develop a fundamental understanding for the mechanobiology of tissue-engineered materials. The regulatory and financial limitations on performing large animal experiments demonstrate the need for accurate and predictive in silico models. Furthermore, in silico models together with experiments provide deeper insights into the growth and remodeling process, which are necessary to optimize the maturation process and implant design. In this work, we relied on in vitro experiments; however, the model was developed using a general, thermodynamically consistent approach. Our long-term vision is to apply this model to in situ tissue-engineering problems as well.

The maturation process involves the secretion of extracellular matrix (ECM) which provides structural support for the tissue through a network of protein fibers such as elastin and collagen fibers. Collagen fibers are the primary structural constituent of the tissue. The changes in the mechanical properties of the tissue are driven by the evolution of collagen density and orientation. Consequently, researchers have extensively studied the mechanisms governing collagen fiber evolution (Huang and Yannas 1977; Wyatt et al. 2009; Siadat and Ruberti 2023; Ruberti and Hallab 2005). During the maturation process, internal stresses develop within the biological tissue. Biological tissues achieve equilibrium at a certain level of stress, called homeostatic stress, in a phenomenon known as tensional homeostasis (Stamenović and Smith 2020). Experimental investigations by Eichinger et al. (2021) demonstrated that the homeostatic stress level depends on collagen density. The process of regulating tissue homeostatic stress drives changes in tissue shape and the reorientation of collagen fibers. ECM homeostasis has been thoroughly investigated in the context of cancer research. Investigations by Paszek et al. (2005) revealed that the stiffness of the ECM influences cell contractility and the tensional homeostatic behavior of the tissue. These findings align with the results obtained from studies on soft tissue constructs by Eichinger et al. (2021). In another study, Cox and Erler (2011) investigated the influence of homeostatic conditions on ECM remodeling.

Researchers have explored various approaches to model biological growth and remodeling. Common approaches are (i) kinematic-based models (Rodriguez et al. 1994), (ii) constrained mixture models (Humphrey and Rajagopal 2002), and (iii) agent-based models (Bonabeau 2002; An et al. 2009). Kinematic models can be classified as either macroscopic or microscopic. Macroscopic models adopt a phenomenological approach, while microscopic models focus on describing the mechanobiological behavior of the material at the cellular level. Among the extensive literature on microscopic models, the following papers are particularly relevant to our work (Zahalak et al. 2000; Alisafaei et al. 2019; Shenoy et al. 2016; Alisafaei et al. 2025). Macroscopic models are especially advantageous for computing structural problems due to their computational efficiency. Constrained mixture models require defining material parameters for each tissue constituent such as smooth muscle cells, elastin, and collagen fibers. Experimental identification of these parameters is highly challenging.

In this work, we propose a kinematic-based macroscopic framework. Biological growth occurs either as a change in shape, which is referred to as volumetric growth, or as changes in the densities of tissue constituents. Both forms alter the total mass of the system. Kinematic models describe volumetric growth using the multiplicative split of the deformation gradient into elastic and inelastic growth parts (Rodriguez et al. 1994). The growth part is called the growth tensor. This approach has been applied to various problems, including studying growth mechanisms in heart valves (Oomen et al. 2018) and in-stent restenosis (Manjunatha et al. 2022, 2023). Reviews on growth models and their applications can be found in Ambrosi et al. (2011); Kuhl (2014); Eskandari and Kuhl (2015); Ambrosi et al. (2019). Early modeling approaches introduced heuristic assumptions about growth direction by prescribing the orientation of the growth tensor based on prior knowledge about growth orientation. While these approaches are effective in many cases, and can give excellent results as demonstrated in Emmert et al. (2018); Drews et al. (2020). These methods may face limitations when applied to general constrained boundary conditions, potentially leading to unphysical predictions (Braeu et al. 2017, 2019; Soleimani et al. 2020; Lamm et al. 2022). To address these limitations, Braeu et al. (2019) developed an anisotropic growth model based on the concept of homeostatic stress, while Soleimani et al. (2020) introduced a stress-driven anisotropic growth framework. Another approach was proposed in Lamm et al. (2022) which derives growth tensors from the homeostatic stress surface defined in the principal stress space. This ensures thermodynamic consistency and accurately describes the evolution of residual stresses. Later Holthusen et al. (2023) developed a two-surface model for growth and remodeling. Additionally, the physics-based inelastic Constitutive Artificial Neural Networks framework (Holthusen et al. 2025) has demonstrated promising results in modeling volumetric growth.

Despite extensive research in biomechanics on modeling growth and remodeling, most models focus on native tissues. Studies addressing modeling the tissue-engineering process are limited. Szafron et al. (2019) demonstrated that numerical models can significantly improve the design of tissue-engineered implants; however, the study did not demonstrate thermodynamic consistency. Later Drews et al. (2020) used a very similar theoretical framework to model the reversal of stenosis. Similarly, Loerakker et al. (2013, 2016), Sanders et al. (2016) and Emmert et al. (2018) provided valuable insights into heart valve mechanobiology. However, these models neglected volumetric growth and lacked proof of satisfying the laws of thermodynamics. The constitutive model introduced in Sesa et al. (2023) to model the maturation process of textile-reinforced tissue-engineered implants considers collagen density evolution to be driven by biochemical and mechanobiological factors. The strain energy in collagen fibers was chosen as the driving factor for the mechanobiological stimulation, ensuring the thermodynamic consistency of the model. However, the model neglected volumetric growth and fiber reorientation. Consequently, the cumulative increase in internal stresses driven by shape evolution was neglected. A more realistic model should consider volumetric growth, as well as the evolution of collagen density and orientation. Modeling these interdependent phenomena requires constructing a coupled system of evolution equations to fully capture the behavior of the tissue. The models for collagen densification (Sesa et al. 2023), as well as volumetric growth and remodeling (Holthusen et al. 2023) provide a solid theoretical basis for the construction of a generalized and thermodynamically consistent growth model for tissue-engineered implants.

In this contribution, we introduce a predictive model describing how the mechanical properties of biological tissue evolve throughout the maturation process. The model describes the underlying phenomena of collagen fiber build-up and the shape evolution. During the maturation process, biological tissues undergo significant changes in their mass, volume, and mechanical properties. This unique behavior renders models developed to describe the evolution of native tissues unsuitable. Existing models developed to optimize the implant’s design such as Szafron et al. (2019), are valuable. However, the development of objective and thermodynamically consistent constitutive models may further enhance the accuracy of predictive models for tissue-engineering processes. These considerations motivated us to develop a more general and thermodynamically consistent approach.

This work presents significant distinctions from our previous work in (Holthusen et al. 2023; Sesa et al. 2023). First, unlike our prior work in Sesa et al. (2023), which modeled collagen density evolution in textile-reinforced implants without considering collagen reorientation and volumetric growth, this study incorporates both of these crucial factors to simulate the behavior of unreinforced tissues. This distinguishes our approach from Holthusen et al. (2023), where collagen density evolution is neglected. Another distinction is our focus here on modeling tissues that initially lack any collagen content and computing their evolution for a period of four weeks, compared to 1–2 days in Holthusen et al. (2023). These considerations require for the first time defining the homeostatic stress as a function of collagen density, rather than a constant (Lamm et al. 2022; Holthusen et al. 2023). Additionally, we introduce a one-surface approach with two pseudo-potentials, which combine the benefits of a two-surface approach in separately describing the evolution of the matrix and collagen parts (Holthusen et al. 2023), while eliminating the need to define two homeostatic stress parameters for the matrix and collagen parts, which are experimentally challenging to identify.

The next sections are organized as follows. Section 2, introduces the theoretical aspects of the model, including constitutive laws and evolution equations. Then, Section 3 provides a concise description of the finite element implementation. In Section 4, we compute two structural examples. The first example mimics the experimental setup for the in vitro maturation of uniaxially constrained construct. Experimental data are used to identify material parameters and validate the numerical model. In the second example, we computed the evolution of a cruciform-shaped construct subjected to biaxial constraints and biaxial load perturbation. The aim is to study the capabilities of our framework in describing structures under complex loading conditions. Then, Section 5 discusses the results and the limitations of the model. Finally, in Section 6, we present our conclusion and outlook for future studies.

## Continuum mechanics model

This section presents a constitutive modeling framework based on continuum mechanics to describe the evolution of biological material during maturation. To ensure the simplicity of the model, we focus only on constituents and mechanisms that significantly affect the material’s mechanical response. A common approach to model soft collagenous tissues is to split the total Helmholtz free energy into an isotropic matrix part and an anisotropic collagen part. The validity of this modeling approach for tissue-engineered constructs was confirmed in Sesa et al. (2023), which measured the stress–strain response of biological constructs and the corresponding alterations in collagen density at different time points during the maturation process. Building on this work, we propose here a model that better describes the evolution of the tissue’s mechanical properties during the maturation process. The model considers three phenomena as the main factors influencing the mechanical response: i) collagen density distribution, ii) collagen orientation, and iii) volumetric growth.

We start by introducing the kinematic relations and the relevant balance equations. Then, we derive the Clausius–Duhem inequality. Next, we extend our equations to account for the decomposition of the Helmholtz free energy into an isotropic matrix and anisotropic collagen parts. Afterward, we introduce the evolution equations describing volumetric growth, collagen density evolution, and fiber reorientation. Finally, we define specific Helmholtz free energy functions chosen for the matrix and collagen parts.

### Kinematics

The first step in constructing our continuum mechanics model is to define the kinematic relations. In a three-dimensional continuum body, the function $\phi$ maps between the reference configuration $\Omega _{0}$ at time $t = 0$ and the current configuration $\Omega$ at time *t*. The position vector for a material particle in the reference configuration is $\textbf{X}$, and the corresponding position vector in the current configuration is $\textbf{x} = \phi (\textbf{X}, t)$. The mapping from the reference configuration to the current configuration is achieved by applying the deformation gradient tensor $\textbf{F} = \partial \textbf{x}/ \partial \textbf{X}$. Then we obtain the right Cauchy–Green tensor $\textbf{C} = \textbf{F}^{\textrm{T}} \, \textbf{F}$ and the left Cauchy–Green tensor $\textbf{B} = \textbf{F} \, \textbf{F}^{\textrm{T}}$.

The growth of biological materials is an inelastic process. The kinematics of such a process can be described using the multiplicative decomposition of the deformation gradient

1 $$\begin{aligned} \textbf{F} = \textbf{F}_{\textrm{e}} \, \textbf{F}_{\textrm{g}}, \end{aligned}$$

where $\textbf{F}_{\textrm{g}}$ is the growth part, and $\textbf{F}_{\textrm{e}}$ is the elastic part (Rodriguez et al. 1994). In the next step, we define the elastic and growth-related right Cauchy–Green tensors, respectively:

2 $$\begin{aligned} \textbf{C}_{\rm{e}}&:= \textbf{F}^{\rm{T}}_{\rm{e}} \, \textbf{F}_{\rm{e}} = \textbf{F}^{{-T}}_{\rm{g}} \, \textbf{C} \, \textbf{F}_{\rm{e}}, \\ \textbf{C}_{\rm{g}}&:= \textbf{F}^{\rm{T}}_{\rm{g}} \, \textbf{F}_{\rm{g}}. \end{aligned}$$

The tensor $\textbf{F}_{\textrm{g}}$ maps the reference configuration $\Omega _{0}$ to a stress-free intermediate configuration. This intermediate configuration is characterized by its rotational non-uniqueness. The polar decomposition operation $\textbf{F}_{\textrm{g}} = \textbf{R}_{\textrm{g}} \, \textbf{U}_{\textrm{g}}$ splits the tensor into the rotation tensor $\textbf{R}_{\textrm{g}}$ and the right stretch tensor $\textbf{U}_{\textrm{g}}$. From this split, it was identified that $\textbf{U}_{\textrm{g}}$ is uniquely defined, while $\textbf{R}_{\textrm{g}}$ suffers from rotational non-uniqueness. To address this issue, we apply the framework from Holthusen et al. (2023) which proposed performing a pull-back operation of the kinematic quantities and structural tensors to a uniquely defined configuration called the co-rotated intermediate configuration (*cic*). By applying this operation, the right Cauchy–Green tensor in *cic* becomes

3 $$\begin{aligned} \bar{\textbf{C}}_{\textrm{e}}:= \textbf{R}^{-1}_{\textrm{g}} \, \textbf{C}_{\textrm{e}} \, \textbf{R}_{\textrm{g}} = \textbf{U}^{-1}_{\textrm{g}} \, \textbf{C} \, \textbf{U}^{-1}_{\textrm{g}}, \end{aligned}$$

where we refer to quantities defined in *cic* using the notation $(\bar{\phantom{a},})$.

### Balance of linear momentum

When modeling the maturation process of biological tissues, various time scales must be considered. The growth and remodeling processes take place over weeks, while the elastic response occurs on the order of milliseconds. This means that our model can be simplified by applying the slow-growth assumption (Goriely 2017), which means that the balance of mass is satisfied without additional considerations, as demonstrated in Sesa et al. (2023). Consequently, the inertia effect from the added mass is negligibly small, allowing us to consider our system to be quasi-static, and apply the standard balance of linear momentum equation

4 $$\begin{aligned} \textrm{Div}({{\textbf{F}}} \, {{\textbf{S}}}) + {{\textbf{b}}}_{0} = {{\textbf{0}}}, \end{aligned}$$

where ${{\textbf{S}}}$ is the second Piola–Kirchhoff stress, and ${{\textbf{b}}}_{0}$ is the body force vector in the reference configuration.

### Clausius–Duhem inequality

The Clausius–Duhem inequality is

5 $$\begin{aligned} \frac{1}{2} \, {{\textbf{S}}} \cdot \dot{{{\textbf{C}}}} - \dot{\psi } + S_{\textrm{0}} \geqslant 0, \end{aligned}$$

where $S_{0}$ is an additional term that accounts for both the local entropy production and the entropy flux through the boundaries of an open system (Kuhl and Steinmann 2003). The material time derivative is denoted by the shorthand notation $({\cdot})$.

During the maturation process, the build-up of collagen content significantly influence the mechanical response of the material. This necessitates introducing the collagen density in the reference configuration ${\rho }^{0}_{\textrm{co}}$ as an argument in the Helmholtz free energy function ${\psi }$ (Sesa et al. 2023) as expressed here

6 $$\begin{aligned} \psi = \bar{\psi }(\bar{\textbf{C}}_{\textrm{e}}, \bar{\textbf{H}}, {\rho }^{0}_{\textrm{co}}). \end{aligned}$$

The generalized structural tensor $\bar{\textbf{H}}$ defined in Eq. 20 is used to consider the anisotropic behavior of collagen fibers.

The material rate of the Helmholtz free energy becomes

7 $$\begin{aligned} \dot{\psi } = \dot{\bar{\psi }} = \frac{\partial \bar{\psi }}{\partial \bar{\textbf{C}}_{\textrm{e}}} \cdot \dot{\bar{\textbf{C}}}_{\textrm{e}} \, + \, \frac{\partial \bar{\psi }}{\partial \bar{\textbf{H}}} \cdot \dot{\bar{\textbf{H}}} \, + \, \frac{\partial \bar{\psi }}{{\rho }^{0}_{\textrm{co}}} \dot{{{\rho }}}^{0}_{\textrm{co}}, \end{aligned}$$

where $\bar{\textbf{C}}_{\textrm{e}}$, $\bar{\textbf{H}}$ and are defined in the *cic*, and ${\rho }^{0}_{\textrm{co}}$ is defined in the reference configuration.

To reformulate the Clausius–Duhem inequality in Eq. (5), we introduce the growth-related velocity gradient in the *cic*

8 $$\begin{aligned} \bar{\textbf{L}}_{\textrm{g}} = \dot{\textbf{U}}_{\textrm{g}} \, \textbf{U}^{-1}_{\textrm{g}}. \end{aligned}$$

Using the definition of $\bar{\textbf{L}}_{\textrm{g}}$ in Eq. (8), we can formulate the following rate quantities

9 $$\begin{aligned} & \dot{\bar{\textbf{C}}}_{\textrm{e}} = {\textbf{U}}^{-1}_{\textrm{g}} \, \dot{\textbf{C}} \, \textbf{U}^{-1}_{\textrm{g}} - \bar{\textbf{C}}_{\textrm{e}} \, \bar{\textbf{L}}_{\textrm{g}} - \bar{\textbf{L}}^{T}_{\textrm{g}} \, \bar{\textbf{C}}_{\textrm{e}}, \end{aligned}$$

10 $$\begin{aligned} & \dot{\bar{\textbf{H}}} = \textbf{U}_{\textrm{g}} \, \dot{\textbf{H}} \, \textbf{U}_{\textrm{g}} + \bar{\textbf{L}}_{\textrm{g}} \, \bar{\textbf{H}} + \bar{\textbf{H}} \, \bar{\textbf{L}}^{T}_{\textrm{g}}. \end{aligned}$$

From the Coleman–Noll procedure (Coleman and Noll 1963), we get the following expression for the second Piola–Kirchhoff stress

11 $$\begin{aligned} \textbf{S} = 2 \, \textbf{U}^{-1}_{\textrm{g}} \, \frac{\partial \bar{\psi }}{\partial \bar{\textbf{C}}_{\textrm{e}}} \, \textbf{U}^{-1}_{\textrm{g}}, \end{aligned}$$

and the reduced dissipation inequality becomes

12 $$\begin{aligned} {\mathscr {D}}_{\textrm{red}}:= \underbrace{ \left( \overbrace{2 \, \bar{\textbf{C}}_{\textrm{e}} \, \frac{\partial \bar{\psi }}{\partial \bar{\textbf{C}}_{\textrm{e}}}}^{=: \, \bar{\mathbf {\Sigma }}} \, - \, \overbrace{2 \, \frac{\partial \bar{\psi }}{\partial \bar{\textbf{H}}} \, \bar{\textbf{H}}}^{=: \, \bar{\textbf{Y}}} \right) }_{\bar{\mathbf {\Gamma }}} \cdot \bar{\textbf{L}}_{\textrm{g}} \, - \, \underbrace{ \textbf{U}_{\textrm{g}} \, \frac{\partial \bar{\psi }}{\partial \bar{\textbf{H}}} \, \textbf{U}_{\textrm{g}} }_{=: \, {\textbf{G}}} \cdot \dot{\textbf{H}} \, - \, \frac{\partial \bar{\psi }}{{\partial \rho ^{0}}_{\textrm{co}}} \, \dot{{\rho }}^{0}_{\textrm{co}} \, + \, S_{0} \, \ge 0, \end{aligned}$$

where $\bar{\mathbf {\Sigma }}$ and $\bar{\textbf{Y}}$ are stress-like tensor quantities defined in the *cic*.

By exploiting the symmetry of the tensor $\bar{\mathbf {\Gamma }}$ (Svendsen 2001; Reese 2003; Holthusen et al. 2023), the dissipation inequality in Eq. (12) can be rewritten as

13 $$\begin{aligned} {\mathscr {D}}_{\textrm{red}}:= \bar{\mathbf {\Gamma }} \cdot \bar{\textbf{D}}_{\textrm{g}} \, - \, {\textbf{G}} \cdot \dot{\textbf{H}} \, - \, \frac{\partial \bar{\psi }}{{\partial \rho ^{0}}_{\textrm{co}}} \, \dot{{\rho }}^{0}_{\textrm{co}} \, + \, S_{0} \ge 0, \end{aligned}$$

with $$\bar{\textbf{D}}_{\textrm{g}}:= \textrm{sym}(\bar{\textbf{L}}_{\textrm{g}})$$.

### Extension to a multi-constituent material

The ECM in tissue-engineered materials consists of various constituents, with fibrous constituents such as collagen and elastin fibers significantly affecting the tissue’s mechanical behavior. Elastin fibers are short and dispersed, providing elasticity, while the collagen network tends to be stiffer and exhibits a complex structure that results in highly nonlinear mechanical behavior. Thus, decomposing the material into an isotropic ground matrix and anisotropic collagen fibers is applicable here as demonstrated in Sesa et al. (2023). Based on this, we formulate the total Helmholtz free energy as

14 $$\begin{aligned} \psi = \bar{\psi }(\bar{\textbf{C}}_{\textrm{e}}, \bar{\textbf{H}}, {\rho }^{0}_{\textrm{co}}) \, = \bar{\psi }_{\textrm{m}}(\bar{\textbf{C}}_{\textrm{e}_{\textrm{m}}} ) \, + \, \bar{\psi }_{\textrm{co}}(\bar{\textbf{C}}_{\textrm{e}_{\textrm{co}}}, \bar{\textbf{H}}, {\rho }^{0}_{\textrm{co}}), \end{aligned}$$

where ${\psi }_{\textrm{m}}$ and ${\psi }_{\textrm{co}}$ refer to the Helmholtz free energies for the matrix and collagen parts, respectively. In a similar manner, we decompose the deformation gradient into a matrix part (m) and a collagen part (co) (Holthusen et al. 2023)

15 $$\begin{aligned} \textbf{F}:= \textbf{F}_{\textrm{e}_{\textrm{m}}} \, \textbf{F}_{\textrm{g}_{\textrm{m}}}:= \textbf{F}_{\textrm{e}_{\textrm{co}}} \, \textbf{F}_{\textrm{g}_{\textrm{co}}}. \end{aligned}$$

From the deformation gradients introduced in Eq. (15), we get the corresponding elastic part of the right Cauchy–Green tensor

16 $$\begin{aligned} \textbf{C}_{\textrm{e}_{\textrm{j}}}:= \mathbf {F^{T}}_{\textrm{e}_{\textrm{j}}} \, \textbf{F}_{\textrm{e}_{\textrm{j}}}, \end{aligned}$$

where we refer to the matrix and collagen parts using the index $\textrm{j}=\{\textrm{m}, \textrm{co} \}$. This is then transformed to the *cic* by applying the same procedure presented in Eq. (3), to get

17 $$\begin{aligned} \bar{\textbf{C}}_{\textrm{e}_{\textrm{j}}}:= \textbf{U}^{-1}_{\textrm{g}_{\textrm{j}}} \, \textbf{C} \, \textbf{U}^{-1}_{\textrm{g}_{\textrm{j}}}. \end{aligned}$$

The next step is to introduce the anisotropic behavior of collagen fibers into the constitutive equations using structural tensors. For a vector $\textbf{a}$ which defines the mean orientation of the collagen fibers, we get the following structural tensor

18 $$\begin{aligned} \textbf{M}:= {{\textbf{a}}} \otimes {{\textbf{a}}}. \end{aligned}$$

Then it can be transformed to the *cic* using the transformation process used in (Holthusen et al. 2023; Reese 2003)

19 $$\begin{aligned} \bar{\textbf{M}} = \frac{1}{\textbf{C}_{\textrm{g}_{\textrm{co}}}:\textbf{M}} \, \textbf{U}_{\textrm{g}_{\textrm{co}}} \, \textbf{M} \, \textbf{U}_{\textrm{g}_{\textrm{co}}}. \end{aligned}$$

Furthermore, it is important to consider the dispersion of fiber orientation in collagen bundles. Therefore, we apply the generalized structural tensor formulation from Gasser et al. (2006)

20 $$\begin{aligned} \bar{\textbf{H}}:= \kappa \, {\textbf{I}} \, + \, (1 \, - \, 3 \, \kappa ) \, \bar{\textbf{M}}, \end{aligned}$$

where $\bar{\textbf{H}}$ lies in the *cic*, and $0 \le {\kappa } \le \frac{1}{3}$.

By applying the decomposition into the matrix and collagen parts to the expression of the second Piola–Kirchhoff stress in Eq. (11), we obtain the following expression

21 $$\begin{aligned} \textbf{S} = \sum _{\textrm{j}} \textbf{S}_{\textrm{j}} = 2 \, \sum _{\textrm{j}} \textbf{U}^{-1}_{\textrm{g}_{\textrm{j}}} \, \frac{\partial \bar{\psi }}{\partial \bar{\textbf{C}}_{\textrm{e}_{\textrm{j}}}} \, \textbf{U}^{-1}_{\textrm{g}_{\textrm{j}}}. \end{aligned}$$

Furthermore, the reduced dissipation inequality in Eq. (13) can be rewritten as

22 $$\begin{aligned} {\mathscr {D}}_{\textrm{red}}:= \underbrace{ \bar{\mathbf {\Sigma }}_{\textrm{m}} }_{=: \, \bar{\mathbf {\Gamma }}_{\textrm{m}}} \cdot \bar{\textbf{L}}_{\textrm{g}_{\textrm{m}}} + \underbrace{ \left( \bar{\mathbf {\Sigma }}_{\textrm{co}} - \bar{\textbf{Y}}_{\textrm{co}} + \bar{\mathbf {\Pi }}_{\textrm{co}} \right) }_{=: \, \bar{\mathbf {\Gamma }}_{\textrm{co}}} \cdot \bar{\textbf{L}}_{\textrm{g}_{\textrm{co}}} \, - \, \textbf{G}_{\textrm{co}} \cdot \dot{\textbf{H}} \, - \, \frac{\partial \bar{\psi }}{{\partial \rho ^{0}}_{\textrm{co}}} \, \dot{{\rho }}^{0}_{\textrm{co}} \, + \, S_{0} \, \ge 0, \end{aligned}$$

with $$\bar{\mathbf {\Sigma }}_{\textrm{m}}:= 2 \, \bar{\textbf{C}}_{\textrm{e}_{\textrm{m}}} \, \frac{\partial \bar{\psi }}{\bar{\textbf{C}}_{\textrm{e}_{\textrm{m}}}}$$, $$\bar{\mathbf {\Sigma }}_{\textrm{co}}:= 2 \, \bar{\textbf{C}}_{\textrm{e}_{\textrm{co}}} \, \frac{\partial \bar{\psi }}{\bar{\textbf{C}}_{\textrm{e}_{\textrm{co}}}}$$, $$\bar{\textbf{Y}}_{\textrm{co}}:= 2 \, \frac{\partial \bar{\psi }}{\partial \bar{\textbf{H}}} \, \bar{\textbf{H}}$$, and $$\bar{\mathbf {\Pi }}_{\textrm{co}}:= 2 \, \frac{\partial \bar{\psi }}{\partial \bar{\textbf{H}}} \cdot (\bar{\textbf{H}} \otimes \bar{\textbf{H}})$$.

### Evolution equations

The next step is to introduce a set of evolution equations to describe the material behavior during the maturation process. The three phenomena considered are i) volumetric growth, ii) collagen density evolution, and iii) collagen reorientation. These phenomena and their interactions are the main driving factors influencing the evolution of the tissue’s mechanical behavior. Therefore, constructing a coupled system of evolution equations is essential for a realistic description of the material response.

#### Volumetric growth

The term volumetric growth is often used to describe the biological process of morphogenesis. This process involves a change in the shape of the tissue, and consequently the build-up of internal stresses. In this work, volumetric growth is modeled using the concept of homeostatic surface, which was first introduced by Lamm et al. (2022). It was later extended by Holthusen et al. (2023) to model anisotropic soft collagenous tissues. The study compared a one-surface and two-surface approach. The numerical investigations in Holthusen et al. (2023) showed that a two-surface provides higher accuracy in modeling growth and remodeling. However, it requires introducing two material parameters for the homeostatic stresses in the matrix and collagen parts, whereas a one-surface model requires only one parameter for the total homeostatic stress.

Experimental Identification of the homeostatic stresses for each constituent can be highly challenging. Hence, no relevant investigations have been found in the literature. Therefore, we chose a one-surface approach. However, we developed a *non-associative* growth model with two pseudo-potentials $g_{\textrm{m}}$ and $g_{\textrm{co}}$ for the matrix and collagen constituents, respectively. This approach makes it is possible to define separate evolution equations for the matrix and collagen parts without the need to introduce additional material parameters for the homeostatic stress of each constituent. We examined various formulations for the homeostatic surface, and chose this formulation as it was able to describe tensional homeostasis:

23 $$\begin{aligned} \phi _{\textrm{g}}:= \frac{1}{J^{2}} \textrm{tr}(\textbf{Y}_{\textrm{g}}^{2}) + \beta _{\textrm{g}} - (2 \, \sigma _{\textrm{g}} \, - \, \frac{1}{J^{2}} \textrm{tr}(\textbf{Y}_{\textrm{g}}))^{2}, \end{aligned}$$

where

24 $$\begin{aligned} \tilde{\varvec{\tau }} = \textbf{U}^{-1}\, \underbrace{\left( \textbf{U}_{\textrm{g}_{\textrm{m}}} \, \bar{\mathbf {\Sigma }}_{\textrm{m}} \, \textbf{U}^{-1}_{\textrm{g}_{\textrm{m}}} + \textbf{U}_{\textrm{g}_{\textrm{co}}} \, \bar{\mathbf {\Sigma }}_{\textrm{co}} \, \textbf{U}^{-1}_{\textrm{g}_{\textrm{co}}} \right) }_{=: \textbf{Y}_{\textrm{g}}} \, \textbf{U}, \end{aligned}$$

is the co-rotated Kirchhoff stress tensor. $\tilde{\varvec{\tau }}$ lives in the co-rotated configuration *crc* and has the same eigenvalues as the Kirchhoff stress ${\varvec{\tau }}$. Furthermore, $\textbf{Y}_{\textrm{g}}$ has the same eigenvalues as ${\varvec{\tau }}$. Therefore, the homeostatic stress function $\phi _{\textrm{g}}$ in Eq. (23) is formulated as a function of $\textbf{Y}_{\textrm{g}}$.

Investigations on homeostatic stress shows that its value is influenced by the composition of biological tissue. An extensive investigation by Eichinger et al. (2021) on soft collagenous tissues found a linear correlation between collagen density and homeostatic stress. This relationship arises because cells sense and respond to the mechanical properties of their local extracellular matrix, primarily determined by collagen density, through mechanotransduction pathways (Humphrey et al. 2014). It is important to consider that homeostasis is regulated locally (Humphrey and Cyron 2022). These considerations are especially important when modeling the maturation process because the initial state of our system is collagen-free. In this situation considering a constant value for the homeostatic stress $\sigma _{g}$ is far from accurate. Therefore, we define the homeostatic stress as a function of the local collagen density in the ${\rho }_{\textrm{co}}$ using the following expression:

25 $$\begin{aligned} \sigma _{g} \, = \, \sigma _{\textrm{g, 0}} \, \left( 1 \, + \, r_{\textrm{1}} \, \frac{{\rho }_{\textrm{co}}}{\rho _{\textrm{co, f}}} \right) . \end{aligned}$$

Here we introduced the initial homeostatic stress $\sigma _{\textrm{g, 0}} \, \mathrm {[MPa]}$ and the coupling coefficient $r_{\textrm{1}} \, \mathrm {[MPa]}$ as additional material parameters. The value of $r_{\textrm{1}}$ determines the influence of collagen density on the homeostatic stress $\sigma _{g}$. The collagen density in the current configuration is ${\rho }_{\textrm{co}} = \frac{1}{J} \, {\rho }^{0}_{\textrm{co}}$ and $\rho _{\textrm{co, f}}$ is an additional parameter representing the average collagen density in the specimen measured at the end of the maturation process.

The next step is to define the pseudo-potentials for each constituent. For the matrix part, we use the following Rankine-like function

26 $$\begin{aligned} g_{\textrm{m}}:= {\left\{ \begin{array}{ll} \frac{1}{J} \textrm{tr}(\textbf{Y}_{\textrm{g}_{\textrm{m}}}), & \frac{1}{J^{2}} \, \textrm{tr}(\textbf{Y}_{\textrm{g}_{\textrm{m}}}^{2}) + \beta _{\textrm{g}} = 0\\ \frac{1}{J} \textrm{tr}(\textbf{Y}_{\textrm{g}_{\textrm{m}}}) + \sqrt{\frac{1}{J^{2}} \textrm{tr}(\textbf{Y}_{\textrm{g}_{\textrm{m}}}) + \beta _{\textrm{g}} }, & else. \end{array}\right. } \end{aligned}$$

Case differentiation is introduced to avoid numerical instabilities that occur when the value under the square root approaches zero.

The growth potential in the collagen part is

27 $$\begin{aligned} g_{\textrm{co}}:= \frac{1}{J} \frac{1}{\bar{\textbf{C}}_{\textrm{e}_{\textrm{co}}}:\bar{\textbf{M}}} \, \bar{\mathbf {\Gamma }}_{\textrm{co}}: \textrm{sym}(\bar{\textbf{C}}_{\textrm{e}_{\textrm{co}}} \bar{\textbf{M}}). \end{aligned}$$

From these potential functions, we can compute the growth directions in each constituent as

28 $$\begin{aligned} \textbf{N}_{\textrm{g}_{\textrm{j}}}:= \frac{\partial g_{\textrm{j}} }{\partial {\bar{\mathbf {\Sigma }}}_{\textrm{j}} }, \end{aligned}$$

and the normalized evolution equation is

29 $$\begin{aligned} \bar{\textbf{D}}_{\textrm{g}_{\textrm{j}}} = \dot{\gamma _{\textrm{g}}} \, \frac{\textbf{N}_{\textrm{g}_{\textrm{j}}}}{ \Vert \textbf{N}_{\textrm{g}_{\textrm{j}}} \Vert }, \end{aligned}$$

where $\dot{\gamma _{\textrm{g}}}$ is the growth multiplier.

The evolution equations are based on the concept developed by Perzyna (1971) to model visco-plastic problems. The growth multiplier is a rate quantity, which depends on the stress deviation from the homeostatic surface (Lamm et al. 2022; Holthusen et al. 2023). The growth multiplier $\dot{\gamma _{\textrm{g}}}$ is computed by solving the following equation

30 $$\begin{aligned} \phi _{\textrm{g}} \, - \, ( 4 \, \sigma _{\textrm{g}}^{2} \, - \, \beta _{\textrm{g}}) (\eta _{\textrm{g}} \, \gamma _{\textrm{g}})^{v_{\textrm{g}}} = 0, \end{aligned}$$

where $\eta _{\textrm{g}}$ is the growth relaxation time, and $v_{\textrm{g}}$ is an additional parameter that describes the nonlinearity of the rate-dependent response.

#### Collagen density evolution

The next step is to introduce evolution equations describing changes in collagen density. Here we apply the evolution equations introduced in Sesa et al. (2023). The primary concept in this work is to decompose the collagen evolution into biological and mechanobiological parts as presented in the following equation

31 $$\begin{aligned} \dot{\rho }^{0}_{\textrm{co}} = \dot{\rho ^{0}}_{\textrm{bio}} + \dot{\rho }^{0}_{\textrm{mech}}, \end{aligned}$$

where the quantities $\dot{\rho }^{0}_{\textrm{co}}$, $\dot{\rho }^{0}_{\textrm{bio}}$ and $\dot{\rho }^{0}_{\textrm{mech}}$ are defined in the reference configuration.

This approach was essential to make the model compatible with experimental observations which showed that unconstrained and unloaded specimens show a significant build-up of collagen content during in vitro maturation. Thus, models that describe collagen densification only as a function of mechanical stimulation fail to describe this behavior. Therefore, we introduced a term to describe the biologically driven part of collagen evolution as

32 $$\begin{aligned} \dot{\rho }^{0}_{\textrm{bio}} = a_{1} \, c_{\textrm{cell}} \, \dot{\alpha }_{\textrm{bio}}, \end{aligned}$$

where $a_{1} \, [\upmu \hbox {g} / \textrm{cells}]$ is a coefficient of the biologically driven collagen evolution and $c_{\textrm{cell}}$ is the cell density. The term $\dot{\alpha }_{\textrm{bio}}$ is the time derivative of the S-shaped Weibull cumulative distribution function

33 $$\begin{aligned} \dot{\alpha }_{\textrm{bio}} = \frac{h}{\tau }\, e^{-(t / \tau )^{h}} \, \left( \frac{t}{\tau }\right) ^{h - 1}, \end{aligned}$$

where the parameters $\tau$ and *h* in Eq. (33) control the half-time and the steepness of the curve, respectively. For more details about the specific reasons behind choosing a Weibull cumulative distribution function, we refer the reader to Sesa et al. (2023).

The mechanobiologically driven part is

34 $$\begin{aligned} \dot{\rho }^{0}_{\textrm{mech}} = {\left\{ \begin{array}{ll} a_{2} \, c_{\textrm{cell}} \, f_{\textrm{mech}} \, {\rho }^{0}_{\textrm{co}} \, \frac{({\psi }_{\textrm{co, m}} - {\psi }_{\textrm{crit}})}{{\psi }_{\textrm{crit}}}, & {\psi }_{\textrm{co, m}} \ge {\psi }_{\textrm{crit}},\\ 0, & {\psi }_{\textrm{co, m}} < {\psi }_{\textrm{crit}}. \end{array}\right. } \end{aligned}$$

In Eq. (34), we introduced the coefficient of mechanobiological stimulation $a_{2} \, \mathrm {[mm^{3}/cells/day]}$. Furthermore, ${\psi }_{\textrm{co, m}}$ is the strain energy per unit mass stored in collagen fibers. The parameter ${\psi }_{\textrm{crit}}$ is the threshold for mechanical stimulation. In addition to that, we introduced the exponential decay function $f_{\textrm{mech}}$ which ensures collagen density increase reaches the saturation level at the end of the maturation process. The exponential decay function is described by the following expression

35 $$\begin{aligned} f_{\textrm{mech}} = e^{-({\rho }^{0}_{\textrm{co}} / \rho _{\textrm{th}})}, \end{aligned}$$

where the collagen saturation level is controlled by the parameter $\rho _{\textrm{th}}$.

In our previous paper (Sesa et al. 2023), parameters $a_{1}$, *t*, and *h* were considered to be independent of other parameters in the case of a constrained but unloaded tissue strip. This simplification was possible since volumetric growth was neglected. However, in this model, the evolution of the specimen shape and collagen density influence each other, since the equations are coupled.

#### Collagen fiber reorientation

One of the unique characteristics of living tissues is their ability to reorient their fibrous content to adapt to mechanical loading conditions. Since collagen is the main structural constituent of the tissue, the reorientation of collagen fibers significantly affect the mechanical properties of the implant. Numerical and experimental studies were done to identify the driving factors for collagen reorientation. These findings of these studies can be broadly divided into two groups, namely (i) stress-driven, and (ii) strain-driven fiber reorientation. In this work we choose a stress-driven approach. This choice is motivated by experimental observations which we will discuss in the next section.

In a stress-driven approach, collagen fibers shall reorient themselves toward the direction of the main principal Cauchy stress. It is important to consider that the vector ${{\textbf{a}}}$ that was introduced in Eq. (18) which describes the mean orientation of a collagen bundle is defined in reference configuration, while the Cauchy stress tensor is defined in the current configuration. To overcome this challenge, we follow the approach presented in Holthusen et al. (2023) which takes advantage of the fact that both the Cauchy stress ${\varvec{\sigma }}$ and the co-rotated Kirchhoff stress $\tilde{\varvec{\tau }}$ that we introduced in Eq. (24) share the same eigenvalues. This allows us to write down the evolution equation for fiber reorientation in the co-rotated configuration *crc*. The relation between collagen orientation $\textbf{a}$ in the reference configuration and $\tilde{\textbf{a}}$ in the *crc* is

36 $$\begin{aligned} \tilde{\textbf{a}} = \frac{1}{\sqrt{\textbf{a} \cdot \textbf{C} \cdot \textbf{a}}} \, \textbf{U} \, \textbf{n} \end{aligned}$$

In the next step, we identify the target orientation $\tilde{\textbf{a}}_{\textrm{target}}$ using the following eigenvalue decomposition

37 $$\begin{aligned} \tilde{\varvec{\tau }} = \sum _{i=1}^{3} \tilde{\tau }_{\textrm{i}} \, \textbf{a}_{\tilde{\varvec{\tau }}_{\textrm{i}}} \otimes \textbf{a}_{\tilde{\varvec{\tau }}_{\textrm{i}}} \, = \, \sum _{i=1}^{3} {\tau }_{\textrm{i}} \, \textbf{a}_{\tilde{\varvec{\tau }}_{\textrm{i}}} \otimes \textbf{a}_{\tilde{\varvec{\tau }}_{\textrm{i}}}. \end{aligned}$$

since the eigenvalues of $\tilde{\varvec{\tau }}$ and $\varvec{\tau }$ are equal. The target orientation $\tilde{\textbf{a}}_{target}$ is the eigenvector $\textbf{a}_{\tilde{\varvec{\tau }}_{\textrm{i}}}$ corresponding to the maximum eigenvalue $\tau _{\textrm{i}}$.

The fiber reorientation toward the main principal orientation is defined using the following evolution equation

38 $$\begin{aligned} \dot{\tilde{\textbf{a}}} = \frac{\pi }{2 \, \eta _{\textrm{s}}} (\tilde{\textbf{a}} \times \tilde{\textbf{a}}_{\textrm{target}}) \times \tilde{\textbf{a}}, \end{aligned}$$

where $\eta _{\textrm{s}}$ is the relaxation time for fiber reorientation (Holthusen et al. 2023).

### Specific choices of the Helmholtz free energies

In the previous subsections, we introduced the general form of the Helmholtz free energies. The next step is to define our specific choices of Helmholtz free energies. Finding reasonable choices for the Helmholtz free energy function to describe tissue-engineered collagenous materials was the subject of the work Sesa et al. (2023). In this study, we used measurements of the stress–strain response and the collagen fiber density at various time points during a maturation process that lasted 28 days. These data were then used to choose the Helmholtz free energy function and the corresponding material parameters. The study showed that the matrix part can be described by the following Neo-Hookean material law

39 $$\begin{aligned} {\psi }_{\textrm{m}} = \frac{\mu }{2} \, (\textrm{tr}(\bar{\textbf{C}}_{\textrm{e}_{\textrm{m}}}) -3) \, - \, \mu \, \textrm{ln}(\bar{J}_{\textrm{e}_{\textrm{m}}}) + \frac{\lambda }{4}(\bar{J}^{2}_{\textrm{e}_{\textrm{m}}}- 1 - 2 \textrm{ln}(\bar{J}_{\textrm{e}_{\textrm{m}}})), \end{aligned}$$

where $\bar{J}_{\textrm{e}_{\textrm{m}}} = \sqrt{\textrm{det} ( \bar{\textbf{C}}_{\textrm{e}_{\textrm{m}}} )}$ represents the elastic volumetric change of the matrix part.

The collagen part is modeled using the Fung-type constitutive model (Fung 1990) introduced by Holzapfel et al. (2000), leading to the following term

40 $$\begin{aligned} {\psi }_{\textrm{co}} = \frac{\rho ^{0}_{\textrm{co}}}{\rho _{\textrm{co, f}}} {\left\{ \begin{array}{ll} \frac{k_{1}}{2 \, k_{2}} \, (\exp {[\mathrm {k_{2}} \, \bar{E}_{\textrm{co}}^2 \,]} - 1), & \bar{E}_{\textrm{co}} \ge 0, \\ 0, & \bar{E}_{\textrm{co}}< 0,\end{array}\right. } \end{aligned}$$

where $$\bar{E}_{\textrm{co}} = \textrm{tr}(\mathbf {\bar{\textbf{C}}_{\textrm{e}_{\textrm{co}}}} \, \bar{\textbf{H}}) - 1$$.

The energy function in Eq. (40) is scaled by the relative energy density $\rho ^{0}_{\textrm{co}} / \rho _{\textrm{co, f}}$ to take into account the influence of collagen density evolution. Such a linear correlation between collagen density $\rho ^{0}_{\textrm{co}}$ and energy ${\psi }_{\textrm{co}}$ was identified in Sesa et al. (2023).

## Numerical implementation

In Section 2 we introduced a set of evolution equations defining volumetric growth, collagen density change, and fiber reorientation. Solving this set of ordinary differential equations (ODEs) requires implementing a robust time integration solution scheme. The unknown quantities that need to be solved using our solution scheme are the vectors $\textbf{U}_{\textrm{g}_{\textrm{m}}}$, $\textbf{U}_{\textrm{g}_{\textrm{co}}}$ and $\tilde{\textbf{a}}$ in addition to the growth multiplier $\dot{\gamma _{\textrm{g}}}$ and the collagen density ${\rho }^{0}_{\textrm{co}}$. A system of ordinary differential equations is solved at each Gauss point using a fully implicit temporal integration scheme. To ensure the computational efficiency, we applied the exponential time integration algorithm developed by Vladimirov et al. (2008) to solve finite elastoplasticity problems. Such an integration scheme was later successfully applied to modeling biological growth (Lamm et al. 2022; Holthusen et al. 2023).

Our computational framework is implemented in the finite element program FEAP (Taylor 2020). Choosing FEAP was motivated by the possibility to easily develop user-defined material and element routines. In our implementation of the material and element routines, we relied on the automatic differentiation tool AceGen (Korelc 2002, 2009). Automatic differentiation was utilized to compute the derivative of the residual vector with respect to the internal variables, and the consistent tangent operator.

The numerical examples presented in Section 4 are computed using the continuum finite element formulation Q1STc (Barfusz et al. 2021; Pacolli et al. 2025). Q1STc is an eight-node first-order isoparametric element. The element contains one Gauss point and applies the concept of enhanced assumed strain for hourglass stabilization. This approach eliminates volumetric and shear locking and reduces the computational cost compared to standard finite element formulations.

## Numerical examples

The next step is to evaluate our model’s capabilities. The two examples explored here represent two different experimental setups. In the first example, we simulate the maturation process of a uniaxially constrained soft collagenous tissue. The setup was used by *BioTex* to study the in vitro maturation process. Then, we compare our numerical results with experimental data. In the second example, we study a cruciform-shaped biaxially constrained specimen under load perturbations. The experimental setup was developed by Eichinger et al. (2020), and later numerical computed by Holthusen et al. (2023), which used the experimental data to validate the in silico model. These earlier investigations primarily focused on studying stress homeostasis over 42 hours in tissues with nearly constant collagen content. This differs from our work here, where the focus is on modeling the biomechanical behavior of the tissue over a maturation process that lasts for 28 days.

The contour and vector plots presented in this section were generated using the open-source software ParaView (Ahrens et al. 2005). Curves and histograms were plotted using Matplotlib (Hunter 2007).

### Uniaxially constrained tissue strip

**Fig. 1:**
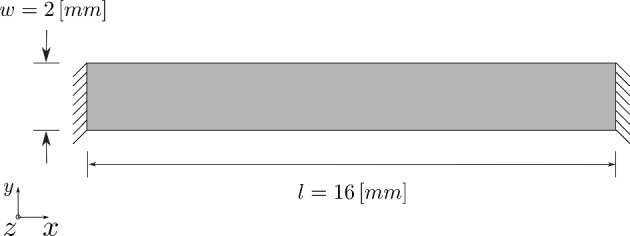
Schematic representation of the boundary value problem for a uniaxially constrained soft tissue construct

In this example, we investigate a uniaxially constrained tissue strip. The initial dimensions and boundary conditions are illustrated in Fig. 1. The schematic shows a tissue strip constrained from both ends. This simple geometry allows us to easily cultivate a large number of samples in a bioreactor. The mechanical and biological characteristics of the cultivated tissues are then investigated. Previous investigations (Sesa et al. 2023), showed that scaling the energy term ${\psi }_{\textrm{co}}$ as shown in Eq. (40) allows us to accurately describe the experimentally measured stress–strain response using a single set of material parameters.

**Table 1:** Material parameters for modeling the maturation of uniaxially constrained collagenous tissue strip

Symbol	Description	Value	Units	Reference
$\lambda$	First Lamé constant of matrix	0.5	$\mathrm {[MPa]}$	Selected
$\mu$	shear modulus of matrix	0.25	$\mathrm {[MPa]}$	Selected
$k_1$	Stiffness-like parameter for the collagen part	0.825	$\mathrm {[MPa]}$	Sesa et al. (2023)
$k_2$	Exponential coefficient for the collagen part	4.0	$[-]$	Sesa et al. (2023)
$\kappa$	Collagen fibers dispersion parameter	0.15	$[-]$	Sesa et al. (2023)
$a_{1}$	biologically driven collagen evolution coefficient	$1 \times 10^{-3}$	$[\upmu \hbox {g} / \textrm{cells}]$	Fitted
$\tau$	Weibull cumulative distribution function half- time	7	$\mathrm {[days]}$	Fitted
*h*	Weibull cumulative distribution function parameter	1.65	$[-]$	Fitted
$a_{2}$	Mechanically driven collagen evolution coefficient	$2.5 \times 10^{-6}$	$\mathrm {[mm^{3}/cells/day]}$	Fitted
${\psi }_{\textrm{crit}}$	collagen fibers Helmholtz free energy per unit mass growth threshold	$2 \times 10^{-5}$	$[\textrm{J}/\upmu \hbox {g}]$	Fitted
${\rho }_{\textrm{th}}$	exponential coefficient controlling collagen density saturation level	6.5	$[\upmu \hbox {g} / \textrm{mm}^{3}]$	Fitted
${\rho }_{\textrm{co, f}}$	Final collagen density	38.7	$[\upmu \hbox {g} / \textrm{mm}^{3}]$	Sesa et al. (2023)
$c_{\textrm{cell}}$	Valvular interstitial cell density	$15 \times 10^{3}$	$\mathrm {[cells/mm^{3}]}$	Hermans et al. (2022)
$\sigma _{\textrm{g, 0}}$	Initial homeostatic stress	0.2	$\mathrm {[MPa]}$	Selected
$r_{\textrm{1}}$	Homeostatic stress coupling coefficient	0.15	$\mathrm {[MPa]}$	Selected
$\beta _{\textrm{g}}$	Stress-like apex parameter	1	$\mathrm {[MPa]}$	Holthusen et al. (2023)
$\eta _{\textrm{g}}$	Volumetric growth relaxation time	50	$\mathrm {[days]}$	Fitted
$\eta _{\textrm{s}}$	Fiber reorientation relaxation time	5	$\mathrm {[days]}$	Fitted
$v_{\textrm{g}}$	Perzyna exponent	1	$[-]$	Holthusen et al. (2023)

**Fig. 2:**
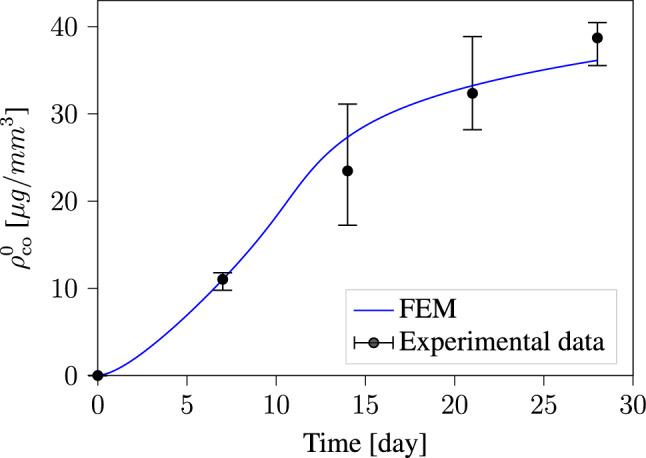
Collagen density evolution during the maturation process. The experimental data are measured after 7, 14, 21, and 28 days of maturation

Initially, the sample does not contain any collagen content. During the in vitro maturation process, we observe the synthesis of ECM which leads to increase in collagen content. Collagen density was measured using a chemical process called * hydroxyproline assay*. Experimental measurements are plotted in Fig. 2. The black dots indicate the mean value of collagen density, and the black bars show the upper and lower range of the measurements. In Sesa et al. (2023), we used these experimental results to identify the parameters $\tau$ and *h* which describe the Weibull cumulative curve. That was possible in our previous investigation because the model neglected volumetric growth and fiber reorientation, which are driven by the evolution of internal stresses. That differs significantly from the model applied here, where our system of equations takes into account the influence of volumetric growth and fiber reorientation on collagen evolution. Consequently, the computed collagen density is influenced by all model parameters listed in Table 1. In this example, we compute the average collagen density $\rho ^{0}_{\textrm{co}}$ for all elements in the computational domain. The computed results are then used to identify the parameters of our evolution equations. The plot in Fig. 2 shows that the model can accurately describe the experimental results.

**Fig. 3:**
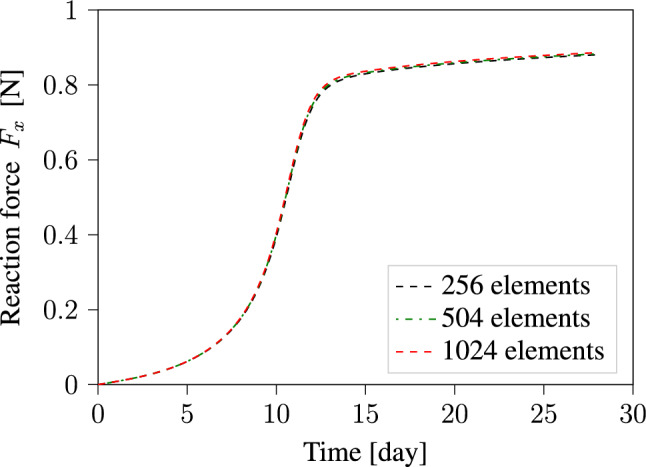
Evolution of the reaction force during the maturation process of uniaxially constrained tissue strip. Computations are performed using three different mesh refinements

To ensure the accuracy of our finite element results, we performed a mesh convergence study. The geometry presented in Fig. 1 was discretized using three different finite element mesh refinements of 256, 504, and 1024 elements. Then we computed the boundary value problem using each mesh refinement and obtained corresponding reaction forces along the x-direction. The reaction forces computed for each mesh refinement are plotted in Fig. 3. The plot shows excellent mesh convergence behavior even with a mesh of only 256 elements. The results we present in this section were computed using a mesh with 1024 elements. In this mesh, the computational domain is discretized along the x, y, and z-directions using 64, 8, and 2 elements, respectively. Such a fine refinement allows us to accurately compute variations in collagen densities and orientations on the local level.

**Fig. 4:**
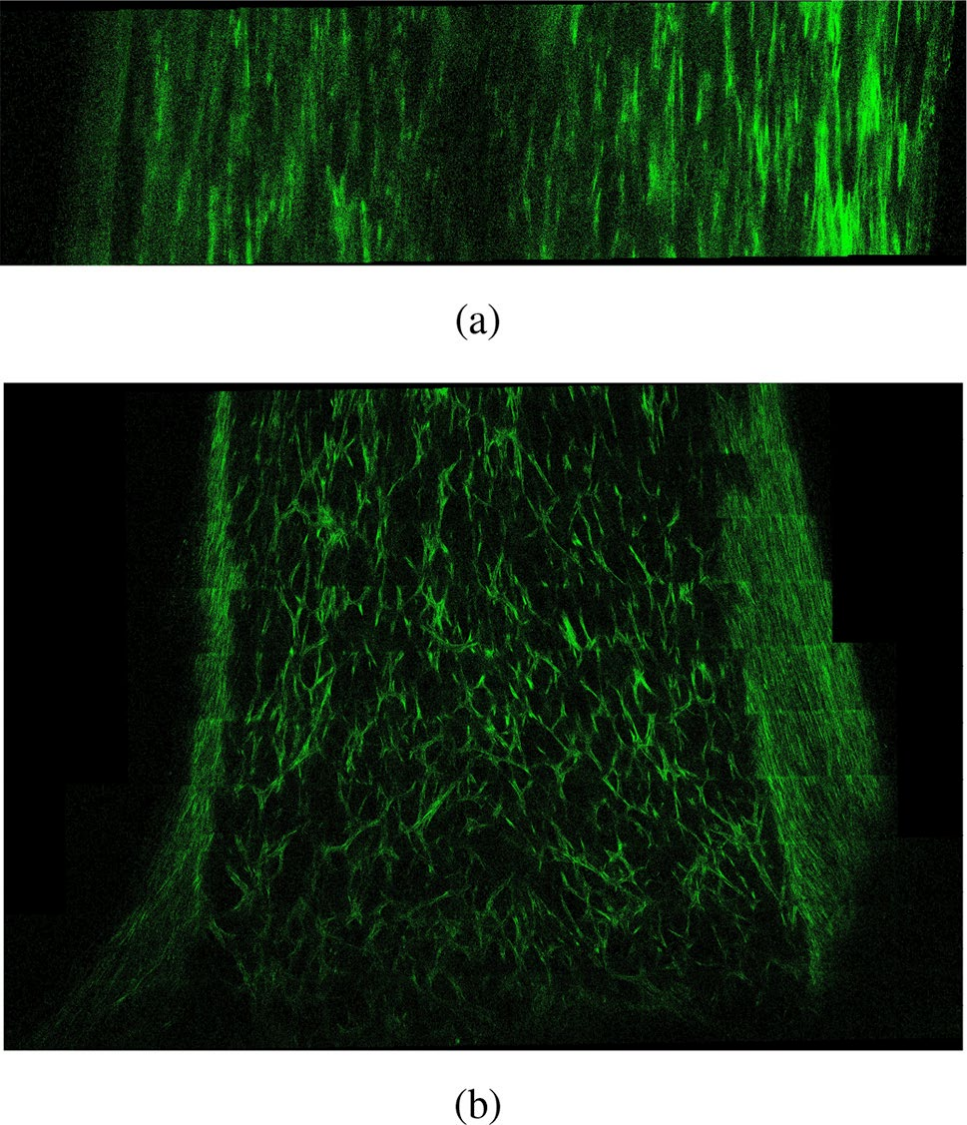
Two-photon microscopy images for the specimen after 15 days of maturation. Collagen fibers are labeled green. (a) Collagen distribution in the middle region, and (b) in the leg region. The positions of each region in the specimen are illustrated in Fig. 8a

**Fig. 5:**
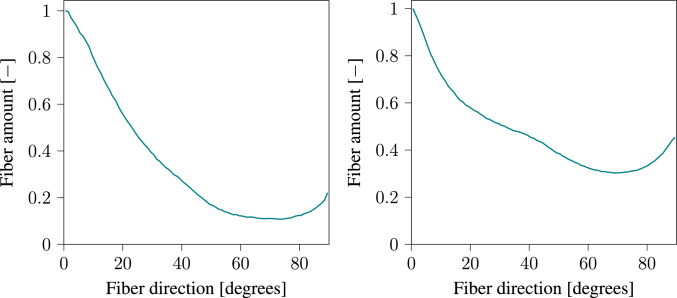
Collagen fiber orientation in the middle region (left) and foot region (right) after 15 days of cultivation

Another aspect investigated during the experiments is the evolution of the specimen shape and collagen fiber orientations. As ECM evolves, internal stresses build up within the tissue. These internal stresses alter the specimen shape, and fiber orientations. Our theoretical formulations in Section 2 are based on the following two hypotheses (i) biological tissues seek to maintain homeostatic stress, and (ii) collagen fibers orient themselves along the main principal stress orientation. Experimental results show that the tissue width *w* shrinks during the maturation. The behavior of collagen fibers depends on their position within the specimen. Here we study the behavior of collagen fibers along the mid-plane (middle region) and at the unixally constrained boundary (leg region). The positions of these two regions are indicated in Fig. 8a. Furthermore, the two-photon microscopy image in Fig. 4a shows that the collagen fibers are uniaxially oriented in the middle region, while Fig. 4b shows that in the leg region, the collagen fibers orientations are highly dispersed. This is clearly demonstrated in Fig. 5, which presents the fiber orientation distributions for both regions.

**Fig. 6:**
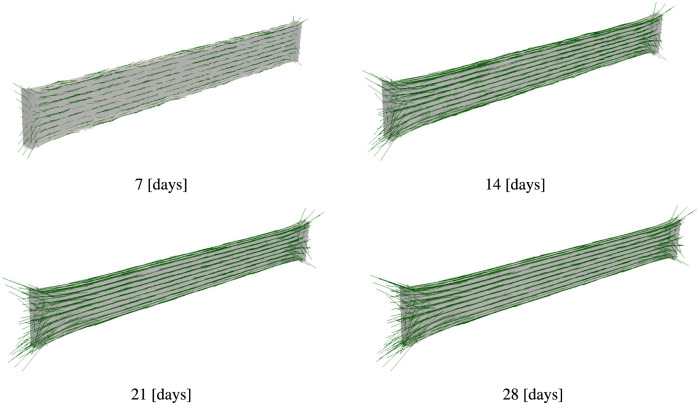
Evolution of collagen fiber density and orientation during the maturation process of the tissue-engineered construct

In our computations, the initial collagen orientations are randomly defined and the initial collagen density is zero. The results in Fig. 6 show that the specimen shape evolves. Furthermore, collagen fibers are visualized in Fig. 6 using green lines, where the length of the fibers refers to the local collagen density at the corresponding element. We can observe that the density increases over time, and collagen fibers reorient themselves. Similar to microscopy images, the results obtained using our model show that collagen fibers in the middle region are uniaxially oriented, while they don’t show the same level of uniaxial orientation in the leg region. These results highlight the ability of the model to describe the physical phenomena observed during the experiments.

**Fig. 7:**
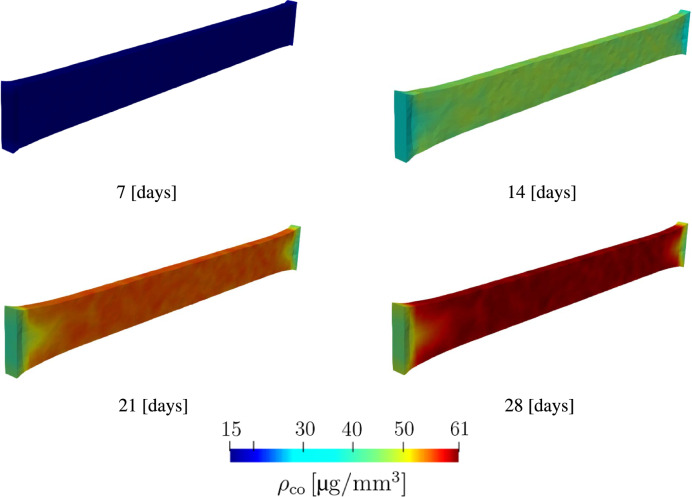
Evolution of collagen density

**Fig. 8:**
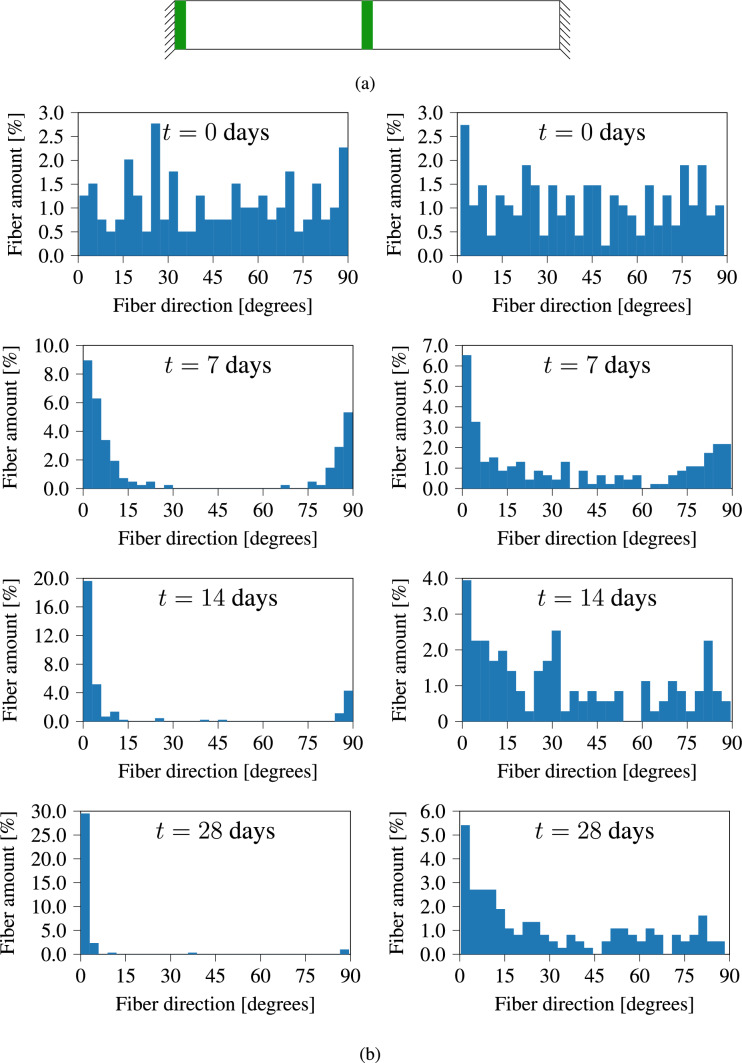
(a) Schematic highlighting regions where collagen orientations are quantified. (b) Collagen fiber orientation at various time points. Figures on the left side fiber distribution at the middle of the specimen, while on the right side is the distribution at the leg region

The distribution of collagen density in the current configuration $\rho _{\textrm{co}}$ is visualized in Fig. 7. We can observe that the variation in collagen density across the sample is small. Such a result is expected since no external load is applied on the sample. Only deformation at the local level occurs due to internal stresses. Furthermore, we performed a quantitative analysis of fiber orientations at the middle and leg regions illustrated in Fig. 8. Collagen fiber orientations are represented at various time steps using histograms. The two charts at the top show the initial distribution of collagen orientations which are randomly defined. The charts on the left show collagen orientations in the middle region, while the charts on the right show the orientations in the leg region. Zero degree represents a fiber orientation along the longitudinal direction. The histograms show clearly that in the middle region, collagen fibers orient themselves along the longitudinal direction, while in the leg region fiber orientation is dispersed. The collagen orientation histograms at day 14 (Fig. 8) closely resemble the fiber orientation distributions observed in Fig. 5 from the in vitro cultivation experiment.

The displacement boundary conditions imposed on the tissue from both sides as illustrated in Fig. 1 lead to the build-up of internal stresses within the material as it seeks to reach homeostatic stress. This behavior manifests itself in the development of reaction forces on these constrained boundaries. The results in Fig. 3 show a steep increase in the reaction force in the first 14 days. After that, the rate of increase in the reaction force slows down significantly. This behavior is influenced by defining the homeostatic stress as a function of collagen density. This makes the model more realistic, as we know from mechanical tests that the build of ECM during the maturation process increases the stiffness of the tissue (Sesa et al. 2023). In our numerical studies, we observed that neglecting this increase in homeostatic stress caused by the increase in collagen content leads to an initial contraction of the specimen width, followed later by an increase in width because lower levels of strain are necessary to maintain the homeostatic stress. This result contradicts experimental observations and exemplify the importance of defining homeostatic stress as a function of collagen density.

### Biaxially constrained cruciform-shaped construct

In the second example, we examine more complex mechanical loading conditions. Here, we investigate a cruciform-shaped construct with biaxial constraints. This setup was used to experimentally investigate tensional homeostasis (Eichinger et al. 2020). Later, Holthusen et al. (2023) framework demonstrated an excellent capability in replicating experimental results. In this work, we go one step further by investigating this boundary value problem in the context of tissue maturation over a period of 28 days. Our goal is to examine the ability of the model proposed here to compute a problem with complex loading conditions.

**Fig. 9:**
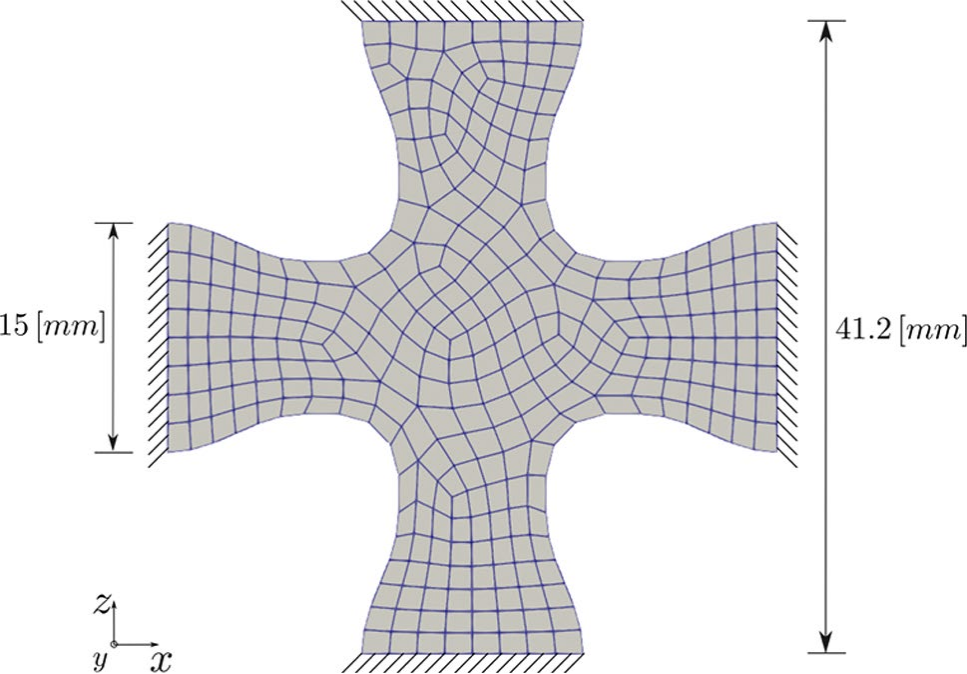
Schematic representation of the boundary value problem for a cruciform-shaped biaxially constrained tissue construct

The boundary value problem that we investigate here is illustrated in Fig. 9. The figure illustrates the biaxial boundary conditions applied to the specimen. We compute the maturation for a period of 28 days. At the time point of $t = 17$ days, a biaxial load perturbation of $20 \%$ is applied. The geometry of the specimen is discretized using 626 cubic elements, with two elements along the thickness (y-direction).

The next step is to define the values of the material parameters. The parameters can be divided into two groups: i) stiffness-like parameters, and ii) parameters describing the evolution equations. For the first group, namely $\lambda$, $\mu$, $k_1$, and $k_2$ we used the values identified in Holthusen et al. (2023). Furthermore, for the parameters $\sigma _{\textrm{g, 0}}$ and $v_{\textrm{g}}$ we applied the values identified in Holthusen et al. (2023). For the remaining parameters, we have selected values that were found to produce reasonable results. The values for $\tau$ and *h* in the Weibull cumulative distribution function, and the final collagen density ${\rho }_{\textrm{co, f}}$ were first identified in Sesa et al. (2023). Regarding the relaxation times $\eta _{\textrm{g}}$ and $\eta _{\textrm{s}}$, our choice was guided by earlier investigations in Holthusen et al. (2023), which showed that $\eta _{\textrm{g}}$ tends to be significantly longer than $\eta _{\textrm{s}}$. As shown in the previous section, the results were fitted using $\eta _{\textrm{s}} = 5$. For the choice of $a_{1}$, $a_{2}$, ${\psi }_{\textrm{crit}}$, and ${\rho }_{\textrm{th}}$ we selected values where both the chemo-biological evolution and mechanobiological stimulation contributed meaningfully to collagen growth. Later in this section, we performed an extensive parameter study, which is discussed in detail in Section 5. The parameter values and the corresponding references are listed in Table 2.

**Table 2:** Material parameters for modeling the maturation of cruciform-shaped biaxially constrained construct

Symbol	Value	Units	Reference
$\lambda$	818	$[\upmu \textrm{N} / \hbox {mm}^{2}]$	Holthusen et al. (2023)
$\mu$	982	$[\upmu \textrm{N} / \hbox {mm}^{2}]$	Holthusen et al. (2023)
$k_1$	3351	$[\upmu \textrm{N} / \hbox {mm}^{2}]$	Holthusen et al. (2023)
$k_2$	14996	$[-]$	Holthusen et al. (2023)
$\kappa$	0.10	$[-]$	Selected
$\sigma _{\textrm{g, 0}}$	22.9	$[\upmu \textrm{N} / \hbox {mm}^{2}]$	Holthusen et al. (2023)
$r_{\textrm{1}}$	10	$[\upmu \textrm{N} / \hbox {mm}^{2}]$	Selected
$a_{1}$	$2 \times 10^{-3}$	$[\upmu \hbox {g} / \textrm{cells}]$	Selected
$\tau$	7	$\mathrm {[days]}$	Selected
*h*	1.65	$[-]$	Selected
$a_{2}$	$5 \times 10^{-6}$	$\mathrm {[mm^{3}/cells/day]}$	Selected
${\psi }_{\textrm{crit}}$	$3 \times 10^{-4}$	$[\textrm{J}/\upmu \hbox {g}]$	Selected
${\rho }_{\textrm{th}}$	10	$[\upmu \hbox {g}/ \textrm{mm}^{3}]$	Selected
${\rho }_{\textrm{co, f}}$	38.7	$[\upmu \hbox {g} / \textrm{mm}^{3}]$	Selected
$c_{\textrm{cell}}$	$15 \times 10^{3}$	$\mathrm {[cells/mm^{3}]}$	Hermans et al. (2022)
$\beta _{\textrm{g}}$	1	$[\upmu \textrm{N} / \textrm{mm}^{2}]$	Holthusen et al. (2023)
$\eta _{\textrm{g}}$	100	$\mathrm {[days]}$	Selected
$\eta _{\textrm{s}}$	5	$\mathrm {[days]}$	Selected
$v_{\textrm{g}}$	1	$[-]$	Holthusen et al. (2023)

**Fig. 10:**
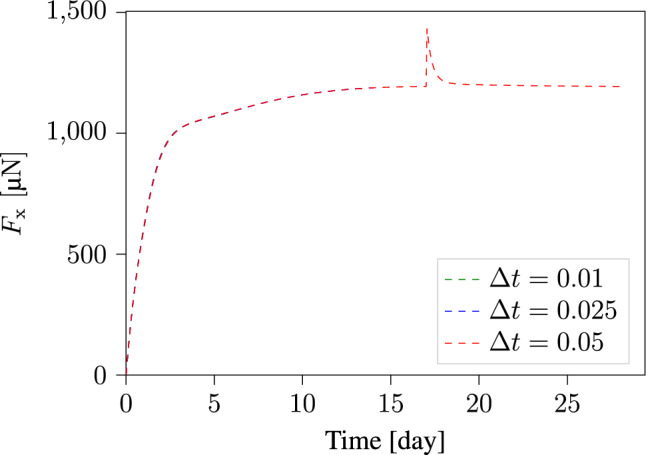
The evolution of reaction forces along the specimen boundaries in the x-direction, computed using three different time-step sizes

We begin by verifying that the choice of time-step size does not substantially affect our results. To investigate this, we performed the computation using three different time-step sizes: 0.01, 0.025, and 0.05 days. We then plotted the evolution of the reaction forces along the x-direction for each time-step size. Plots in Fig. 10 show that the results are almost identical for the three computations, indicating that the results are largely insensitive to the choice of time-step size.

**Fig. 11:**
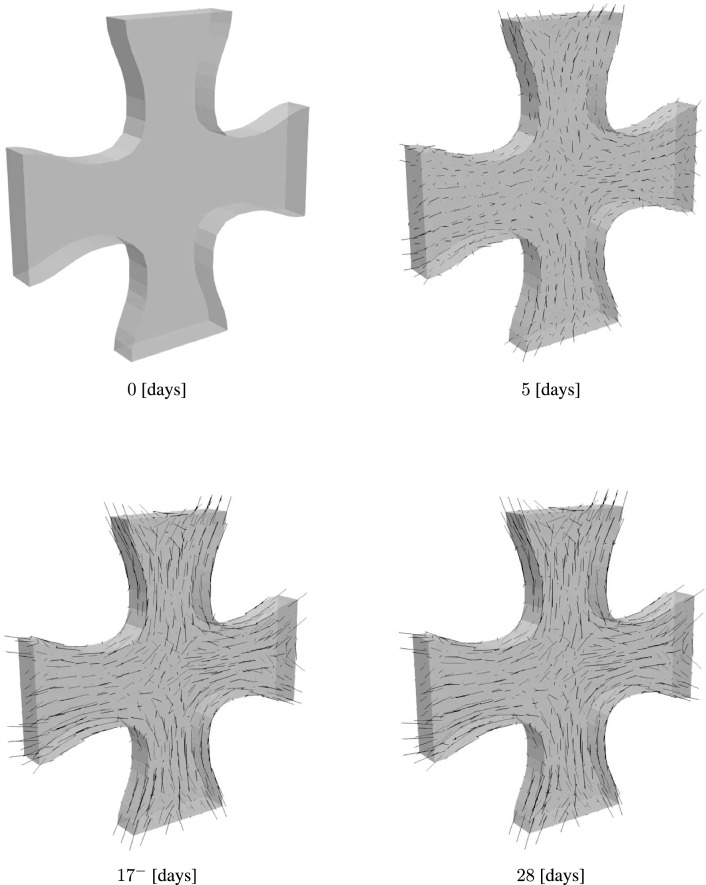
Evolution of collagen fiber density and orientation during the maturation process of a cruciform-shaped biaxially constrained tissue

**Fig. 12:**
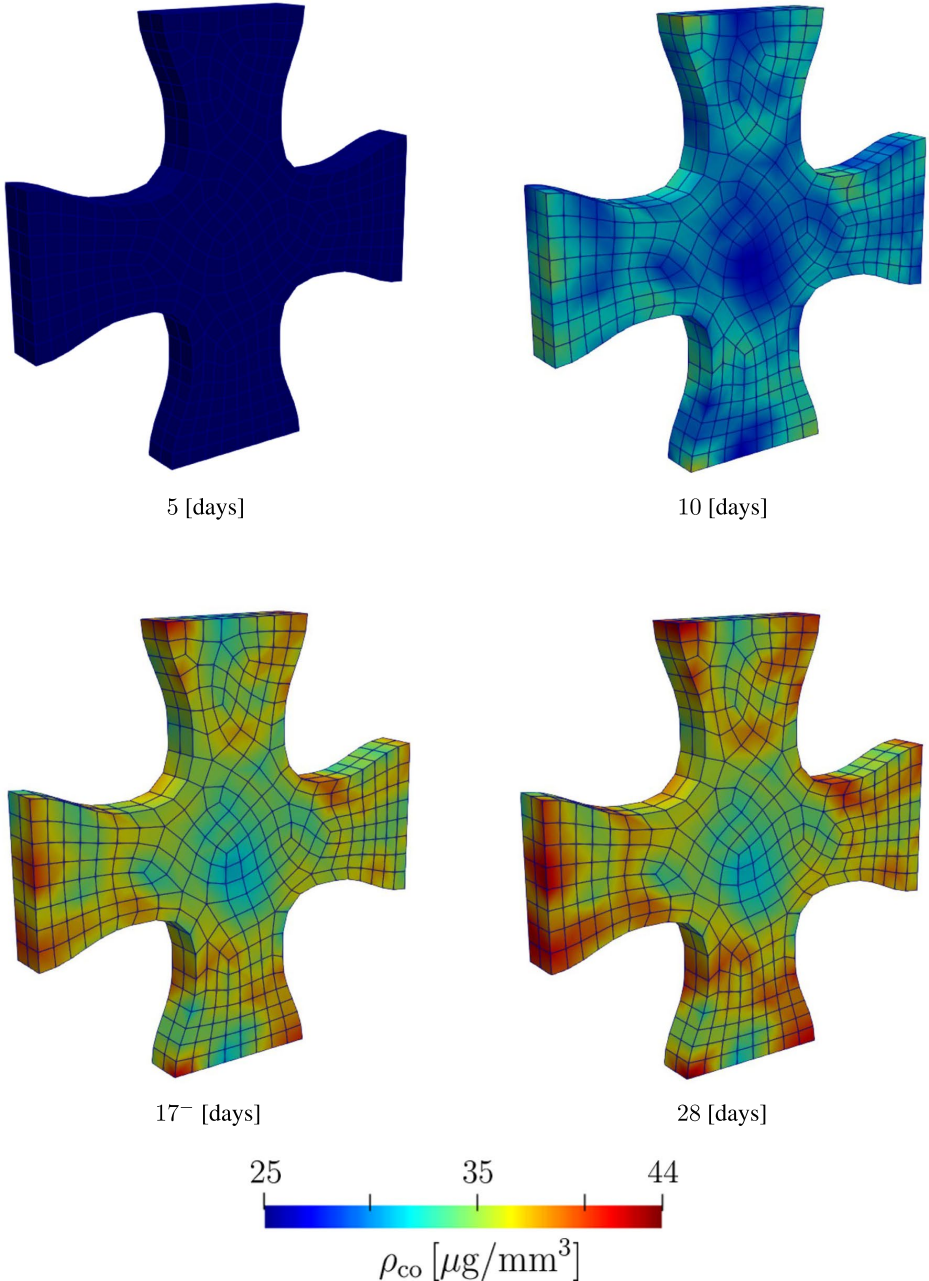
Evolution of collagen density during the maturation process of a cruciform-shaped biaxially constrained tissue

Figure 11 shows the evolution of collagen fibers. Similar to the first example, collagen density is represented using the length of the green lines. The collagen density distribution at these time points is shown in the contour plots in Fig. 12. In this example, we observe that fibers are highly oriented in the axial section, while in the middle region where there is biaxial loading conditions, collagen fibers have a dispersed orientation. Collagen orientations resemble the results in Holthusen et al. (2023). A closer look at the collagen density distribution in Fig. 11, shows lower collagen density in the middle region. This can be explained by Eq. 34 where mechanical stimulation of collagen growth is defined as a function of the energy term ${\psi }_{\textrm{co, m}}$. Fiber dispersion lowers the value of the strain energy ${\psi }_{\textrm{co, m}}$.

**Fig. 13:**
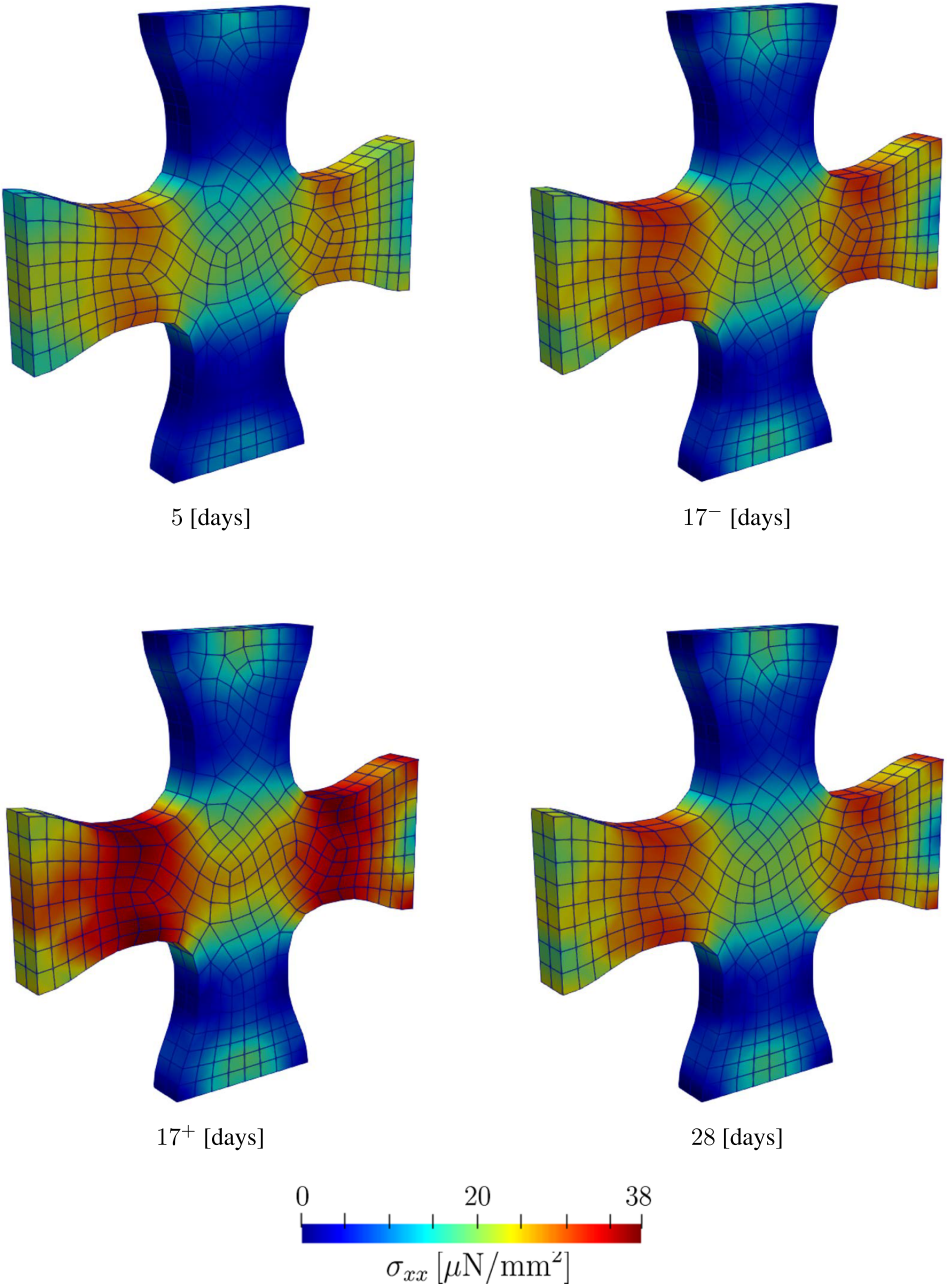
Evolution of the Cauchy stress in the *x* direction at different time steps. $17^{-}$ refers to the last time step before the $20 \%$ load perturbation, and $17^{+}$ refers to first time step after the load perturbation

**Fig. 14:**
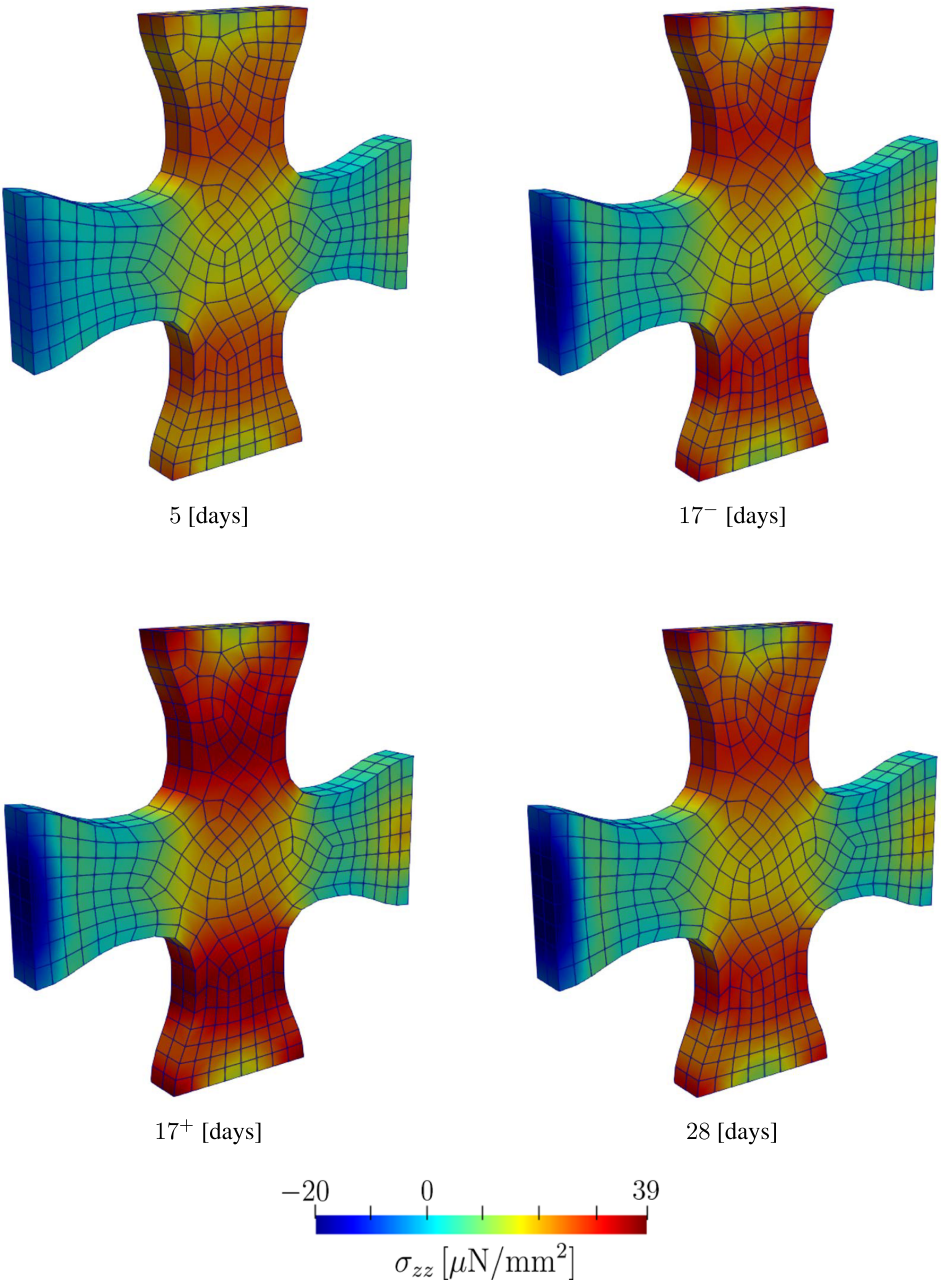
Evolution of the Cauchy stress in the *z* direction at different time steps. $17^{-}$ refers to the last time step before the $20 \%$ load perturbation, and $17^{+}$ refers to first time step after the load perturbation

**Fig. 15:**
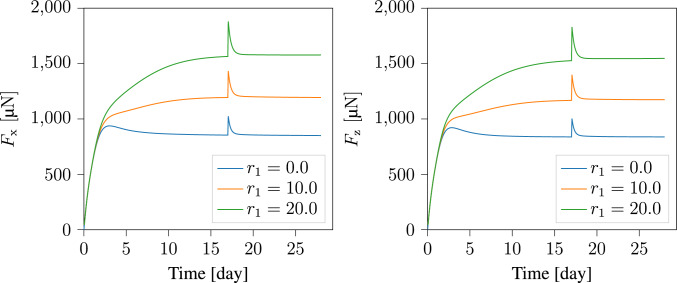
The evolution of reaction forces along the sample boundaries in the (left) x-direction, and (right) z-direction

The stress contours in Fig. 13 and 14 show the evolution of the stresses along the X and Z-directions, respectively. In both figures, we observe a significant increase in the stress following the load perturbation. However, as material returns to the homeostatic stress levels, we observe that stress at time points $17^{-}$ and 28 days are almost identical. $17^{-}$ here refers to the last time point before the load perturbation at $t = 17$. This can be observed in Fig. 15, where the force response returns to the homeostatic level following the load perturbation.

**Fig. 16:**
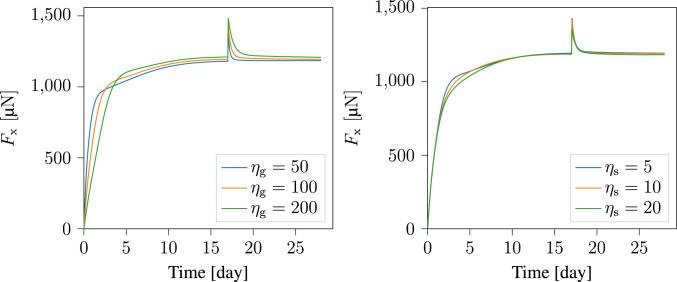
Parameter study for the relaxation time of volumetric growth $\eta _{\textrm{g}}$ (left), and fiber reorientation $\eta _{\textrm{s}}$ (right)

**Fig. 17:**
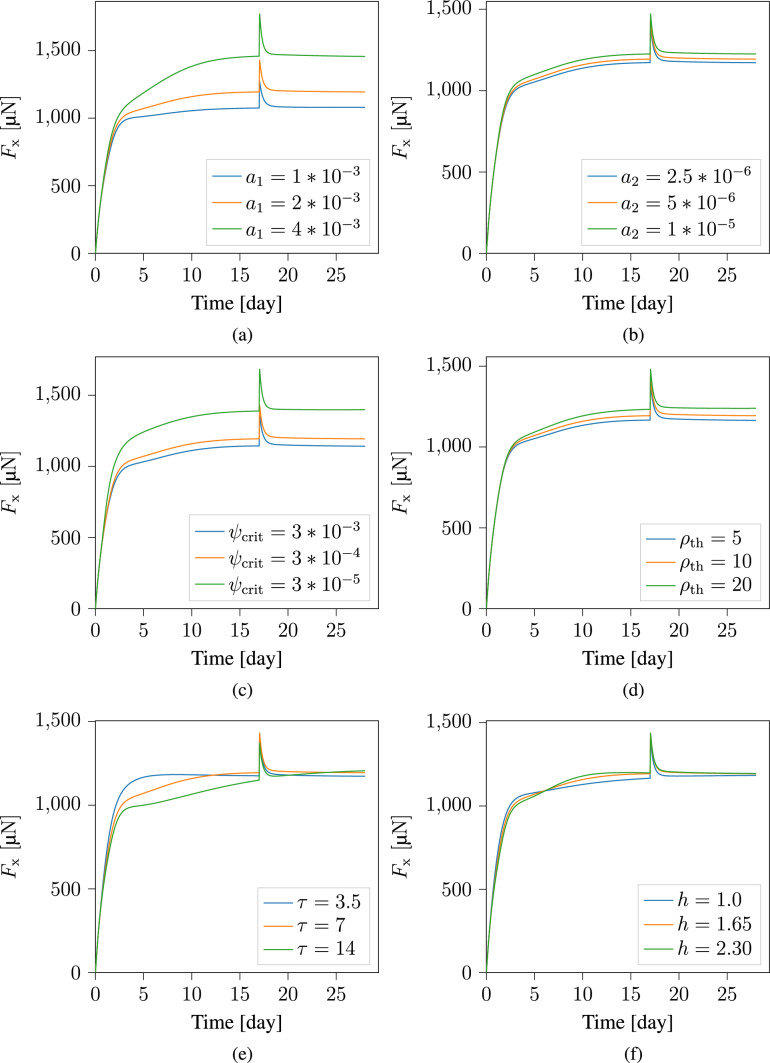
Parameter study for the evolution of reaction forces along the boundary conditions defined in the x-direction. The modeling parameters investigated are: $a_{1}$, $a_{2}$, ${\psi }_{\textrm{crit}}$, ${\rho }_{\textrm{th}}$, $\tau$, and *h*

To better understand the model, we analyzed how various parameters affect the evolution of reaction forces along the boundary conditions. The first parameter we examine is the coupling parameter $r_{\textrm{1}}$, introduced in Eq. 25. In Fig. 15, we present the force response plots along both the x- and z-directions. The plots show nearly identical force responses in both directions. Therefore, in subsequent parametric studies, we present only the force response along the x-direction. Figure 16 explores the effects of the relaxation times $\eta _{\textrm{g}}$ and $\eta _{\textrm{s}}$. Additionally, Fig. 17 examines the influence of the parameters $a_{1}$, $a_{2}$, ${\psi }_{\textrm{crit}}$, ${\rho }_{\textrm{th}}$, $\tau$, and *h* on the mechanical response of the material. These parameters, which describe the evolution of collagen density, were initially introduced in Sesa et al. (2023), where a parameter study on collagen density evolution was presented.

## Discussion

The main objective of this work is to develop a thermodynamically consistent framework for modeling the evolution of soft collagenous tissue shape, as well as collagen density and orientation during the maturation process, without relying on growth tensors with predefined orientations. The model also accounts for the evolution of homeostatic stress, ensuring that key mechanobiological regulatory mechanisms are represented. In Section 4.1, we investigate the in vitro cultivation of uniaxially constrained tissue. A comparison between the fiber orientations predicted by our in silico model (Fig. 8) and those observed experimentally (Fig. 5) shows that the model captures the key mechanobiological phenomena reasonably well.

The second example presented in Section 4.2 builds on the work of Holthusen et al. (2023), who modeled the mechanical response over 42 h. In Holthusen et al. (2023), ten experimental setups were studied, but only three were used to identify material parameters. The model successfully predicted the responses of the remaining setups. Here, we extend the investigation to the tissue’s long-term evolution over 28 days. The same problem was recently studied by Alisafaei et al. (2025), who found that the tissue shows a high level of anisotropy in the axial sections of the specimen, while the middle region is almost isotropic. These results resemble those in Fig. 11, where we observe collagen fibers highly oriented in the axial regions, resulting in a highly anisotropic material, while in the middle region, fiber orientations are dispersed, leading to isotropic behavior. Due to the lack of experimental data on reaction forces for in vitro cultivation over such a long period, we subdivided our parameters into two groups (as explained in Section 4.2). For the first group, which describes the short-term mechanical response, we used parameters from Holthusen et al. (2023), while for the remaining parameters, we selected values that seemed plausible and performed a parameter study.

The parameter studies presented in Fig. 15, 16, and 17 used the baseline parameter set in Table 2, with one parameter varied at a time. The applied displacement at $t\, = \, 17^{+}$ remains constant, leading to $20 \%$ load perturbation in the base case, but this may change with parameter variation. The plots in Fig. 15 show that $r_{\textrm{1}}$ significantly influences the reaction forces. Choosing $r_{\textrm{1}} = 0$ means collagen density does not affect the homeostatic stress. A higher $r_{\textrm{1}}$ value leads to a steeper increase in reaction force, particularly early in the maturation process when densification rate is high. This highlights the importance of considering collagen density influence on the homeostatic stress. Relaxation times $\eta _{\textrm{g}}$ and $\eta _{\textrm{s}}$ also strongly affect material response, as shown in Fig. 16. As expected, shorter relaxation times lead to reaching homeostasis faster. However, changing $\eta _{\textrm{g}}$ slightly affects the final reaction force, while modifying $\eta _{\textrm{s}}$ has minimal influence in this regard. This is because $\eta _{\textrm{g}}$ controls volumetric growth rate, which impacts the mechanically driven densification rate and thus homeostatic stress, whereas $\eta _{\textrm{s}}$, mainly affects collagen reorientation rate.

Next, we investigate collagen density evolution parameters. The coefficient $a_{1}$ is particularly influential, affecting the densification rate in early stages and consequently reaction forces (Fig. 17a). In contrast, Fig. 17b shows that varying the mechanically driven densification coefficient $a_{2}$ has limited effect on the results since deformations are small in this example. A more pronounced effect can be observed under large deformations, as demonstrated in Sesa et al. (2023). Figure 17c shows that the model is highly sensitive to the energy threshold $\psi _{\textrm{crit}}$; lower values lead to significantly higher mechanically driven densification rates, as described by Eq. 34. Additionally, Fig. 17d examines $\rho _{\textrm{th}}$ which also controls the mechanically driven collagen densification rate. Smaller values slow densification when collagen density is high. Finally, we investigate $\tau$ and *h* from the Weibull cumulative distribution function. A higher $\tau$ value slows biologically driven evolution initially, lowering the initial reaction forces. Interestingly, this makes the material less stiff, leading to higher deformation, higher mechanically driven densification, and consequently higher final reaction force (Fig. 17e). In contrast, *h* controls the steepness of the Weibull curve; higher *h* values result in a steeper S-shaped curve, with slower early biologically driven densification rate, followed by a sharp acceleration around time $\tau$. Consequently, reaction forces follow a similar pattern (Fig. 17f).

Regarding the limitations of the model proposed here, the main limitation, as is often the case with phenomenological models, lies in identifying a relatively large number of parameters and validating the model. This process requires conducting a large number of experiments. Additionally, experimental data used for parameter identification exhibit high variability, introducing considerable uncertainty into the model outcomes. To address this issue, Brandstaeter et al. (2021); Wirthl et al. (2023) proposed global sensitivity analysis approaches. In addition to this typical issue in growth modeling, here we face an additional problem: our model was designed based on data from in vitro cultivation experiments. However, the ultimate goal is to apply this model to in situ tissue-engineering problems. Due to the generality of the model we introduce here, the basic mathematical formulations should be valid for in situ maturation. However, extensive and costly in situ experiments are still necessary to validate the model before applying it to clinical applications.

Another major limitation here is that the biologically driven part of collagen density evolution equations is described as a function of time rather than being defined by the chemo-biological factors. This approach was followed by Szafron et al. (2019) to model tissue-engineering process of vascular grafts, and was applied in our earlier work (Sesa et al. 2023). This significantly reduces the number of parameters in the model, and allows us to focus on understanding the mechanobiological factors of the growth process. However, to fully describe the chemo-biological factors driving the tissue-engineering process, a more general chemo-biological approach should be pursued.

## Conclusion and Outlook

This work presents a novel framework for modeling growth and remodeling during the maturation of tissue-engineered implants. A key feature is the explicit treatment of collagen, the main structural constituent in soft biological tissues, whose density and orientation evolve over time. Additionally, volumetric growth is governed by a homeostatic surface approach that ensures compliance with the second law of thermodynamics, thereby providing a more comprehensive and physically consistent model compared to earlier efforts (Lamm et al. 2022; Sesa et al. 2023; Holthusen et al. 2023). A central theoretical advancement is the dependence of homeostatic stress on local collagen content, allowing for spatial variability in tissue composition. Implementing a single homeostatic surface, combined with two growth potentials defined in a non-associative manner, also affords greater flexibility in handling multiple tissue constituents without resorting to multi-surface formulations.

The model was tested in two numerical examples. In the first, the in vitro maturation of a uniaxially constrained tissue strip provided experimental data for parameter identification, verifying the model’s ability to capture collagen density evolution and reorientation with reasonable accuracy. Parameters describing overall tissue stiffness were taken from prior work (Sesa et al. 2023). In the second example, a cruciform-shaped biaxially constrained tissue was subjected to load perturbation, underscoring the model’s capability to handle more complex boundary conditions.

Looking ahead, the proposed modeling framework can be applied to three-dimensional tissue-engineered implants like vascular grafts and heart valves, offering opportunities for more rigorous validation and parameter tuning under clinically relevant conditions. Expanding the model to reinforced biohybrid implants (Boehm et al. 2023) could further illuminate the mechanobiological effects of different scaffold types. Ultimately, embedding this constitutive approach into a multi-physics fluid-solid-growth framework (Figueroa et al. 2009) would enable even more realistic simulations. Another promising direction involves leveraging automatic model discovery techniques (Holthusen et al. 2024, 2025) to refine or generalize the growth and remodeling laws, potentially leading to new insights and clinically relevant predictions.

## References

[CR1] Di Cesare M, Perel P, Taylor S, Kabudula C, Bixby H, Gaziano TA, McGhie DV, Mwangi J, Pervan B, Narula J, Pineiro D, Pinto FJ (2024) The heart of the world. Glob Heart 19(1):3 38273998 10.5334/gh.1288 PMC10809869

[CR2] Amini M, Zayeri F, Salehi M (2021) Trend analysis of cardiovascular disease mortality, incidence, and mortality-to-incidence ratio: results from global burden of disease study 2017. BMC Public Health 21:1–12 33632204 10.1186/s12889-021-10429-0 PMC7905904

[CR3] Yacoub MH, Takkenberg J (2005) Will heart valve tissue engineering change the world?. Nat Clin Pract Cardiovasc Med 2:60–61 16265355 10.1038/ncpcardio0112

[CR4] Pashneh-Tala S, MacNeil S, Claeyssens F (2016) The tissue-engineered vascular graft past, present, and future. Tissue Eng Part B Rev 22(1):68–100 26447530 10.1089/ten.teb.2015.0100 PMC4753638

[CR5] Turner ME, Blum KM, Watanabe T, Schwarz EL, Nabavinia M, Leland JT, Villarreal DJ, Schwartzman WE, Chou T-H, Baker PB et al (2024) Tissue engineered vascular grafts are resistant to the formation of dystrophic calcification. Nat Commun 15(1):2187 38467617 10.1038/s41467-024-46431-4 PMC10928115

[CR6] Uiterwijk M, Smits AI, van Geemen D, van Klarenbosch B, Dekker S, Cramer MJ, van Rijswijk JW, Lurier EB, Di Luca A, Brugmans MC et al (2020) In situ remodeling overrules bioinspired scaffold architecture of supramolecular elastomeric tissue-engineered heart valves. Basic to Trans Sci 5(12):1187–1206 10.1016/j.jacbts.2020.09.011 PMC7775962 33426376

[CR7] Foolen J, Deshpande VS, Kanters FM, Baaijens FP (2012) The influence of matrix integrity on stress-fiber remodeling in 3d. Biomaterials 33(30):7508–7518 22818650 10.1016/j.biomaterials.2012.06.103

[CR8] Hermans L, Van Kelle M, Oomen P, Lopata RG, Loerakker S, Bouten C (2022) Scaffold geometry-imposed anisotropic mechanical loading guides the evolution of the mechanical state of engineered cardiovascular tissues in vitro. Front Bioeng Biotechnol 10:796452 35252127 10.3389/fbioe.2022.796452 PMC8888825

[CR9] Huang C, Yannas IV (1977) Mechanochemical studies of enzymatic degradation of insoluble collagen fibers. J Biomed Mater Res 11:137–154 14968 10.1002/jbm.820110113

[CR10] Wyatt KEK, Bourne JW, Torzilli PA (2009) Deformation-dependent enzyme mechanokinetic cleavage of type i collagen. J Biomech Eng 131 10.1115/1.3078177 PMC2766525 19388774

[CR11] Siadat SM, Ruberti JW (2023) Mechanochemistry of collagen. Acta Biomater 161: 50-62 10.1016/j.actbio.2023.01.025 PMC13270376 36669548

[CR12] Ruberti JW, Hallab NJ (2005) Strain-controlled enzymatic cleavage of collagen in loaded matrix. Biochem Biophys Res Commun 336:483–489 16140272 10.1016/j.bbrc.2005.08.128

[CR13] Stamenović D, Smith ML (2020) Tensional homeostasis at different length scales. Soft Matter 16(30):6946–6963 32696799 10.1039/d0sm00763c

[CR14] Eichinger JF, Grill MJ, Kermani ID, Aydin RC, Wall WA, Humphrey JD, Cyron CJ (2021) A computational framework for modeling cell-matrix interactions in soft biological tissues. Biomech Model Mechanobiol 20(5):1851–1870 34173132 10.1007/s10237-021-01480-2 PMC8450219

[CR15] Paszek MJ, Zahir N, Johnson KR, Lakins JN, Rozenberg GI, Gefen A, Reinhart-King CA, Margulies SS, Dembo M, Boettiger D et al (2005) Tensional homeostasis and the malignant phenotype. Cancer Cell 8(3):241–254 16169468 10.1016/j.ccr.2005.08.010

[CR16] Cox TR, Erler JT (2011) Remodeling and homeostasis of the extracellular matrix: implications for fibrotic diseases and cancer. Disease Model Mech 4(2):165–178 10.1242/dmm.004077 PMC3046088 21324931

[CR17] Rodriguez EK, Hoger A, McCulloch AD (1994) Stress-dependent finite growth in soft elastic tissues. J Biomech 27(4):455–467 8188726 10.1016/0021-9290(94)90021-3

[CR18] Humphrey JD, Rajagopal KR (2002) A constrained mixture model for growth and remodeling of soft tissues. Math Models Methods Appl Sci 12(03):407–430

[CR19] Bonabeau E (2002) Agent-based modeling: Methods and techniques for simulating human systems. Proc Natl Acad Sci 99(suppl–3):7280–7287 12011407 10.1073/pnas.082080899 PMC128598

[CR20] An G, Mi Q, Dutta-Moscato J, Vodovotz Y (2009) Agent-based models in translational systems biology. Wiley Interdiscip Rev Syst Biol Med 1(2):159–171 20835989 10.1002/wsbm.45 PMC3640333

[CR21] Zahalak GI, Wagenseil JE, Wakatsuki T, Elson EL (2000) A cell-based constitutive relation for bio-artificial tissues. Biophys J 79(5):2369–2381 11053116 10.1016/S0006-3495(00)76482-4 PMC1301124

[CR22] Alisafaei F, Jokhun DS, Shivashankar G, Shenoy VB (2019) Regulation of nuclear architecture, mechanics, and nucleocytoplasmic shuttling of epigenetic factors by cell geometric constraints. Proc Natl Acad Sci 116(27):13 200-13 209 10.1073/pnas.1902035116 PMC6613080 31209017

[CR23] Shenoy VB, Wang H, Wang X (2016) A chemo-mechanical free-energy-based approach to model durotaxis and extracellular stiffness-dependent contraction and polarization of cells. Interface Focus 6(1):20150067 26855753 10.1098/rsfs.2015.0067 PMC4686242

[CR24] Alisafaei F, Shakiba D, Hong Y, Ramahdita G, Huang Y, Iannucci LE, Davidson MD, Jafari M, Qian J, Qu C et al (2025) Tension anisotropy drives fibroblast phenotypic transition by self-reinforcing cell-extracellular matrix mechanical feedback. Nat Mater 1–11 10.1038/s41563-025-02162-5 PMC12368865 40128624

[CR25] Oomen PJ, Holland MA, Bouten CV, Kuhl E, Loerakker S (2018) Growth and remodeling play opposing roles during postnatal human heart valve development. Sci Rep 8:1–13 29352179 10.1038/s41598-018-19777-1 PMC5775310

[CR26] Manjunatha K, Behr M, Vogt F, Reese S (2022) A multiphysics modeling approach for in-stent restenosis: theoretical aspects and finite element implementation. Comput Biol Med 150:106166 36252366 10.1016/j.compbiomed.2022.106166

[CR27] Manjunatha K, Schaaps N, Behr M, Vogt F, Reese S (2023) Computational modeling of in-stent restenosis: pharmacokinetic and pharmacodynamic evaluation. Comput Biol Med 167:107686 37972534 10.1016/j.compbiomed.2023.107686

[CR28] Ambrosi D, Ateshian GA, Arruda EM, Cowin S, Dumais J, Goriely A, Holzapfel GA, Humphrey JD, Kemkemer R, Kuhl E et al (2011) Perspectives on biological growth and remodeling. J Mech Phys Solids 59(4):863–883 21532929 10.1016/j.jmps.2010.12.011 PMC3083065

[CR29] Kuhl E (2014) Growing matter: a review of growth in living systems. J Mech Behav Biomed Mater 29:529–543 24239171 10.1016/j.jmbbm.2013.10.009

[CR30] Eskandari M, Kuhl E (2015) Systems biology and mechanics of growth. Wiley Interdiscip Rev Syst Biol Med 7:401–412 26352286 10.1002/wsbm.1312 PMC4600462

[CR31] Ambrosi D, Ben Amar M, Cyron CJ, DeSimone A, Goriely A, Humphrey JD, Kuhl E (2019) Growth and remodelling of living tissues: perspectives, challenges and opportunities. J R Soc Interface 16(157):20190233 31431183 10.1098/rsif.2019.0233 PMC6731508

[CR32] Emmert MY, Schmitt BA, Loerakker S, Sanders B, Spriestersbach H, Fioretta ES, Bruder L, Brakmann K, Motta SE, Lintas V et al (2018) Computational modeling guides tissue-engineered heart valve design for long-term in vivo performance in a translational sheep model. Sci Trans Med 10(440):eaan4587 10.1126/scitranslmed.aan4587 29743347

[CR33] Drews JD, Pepper VK, Best CA, Szafron JM, Cheatham JP, Yates AR, Hor KN, Zbinden JC, Chang Y-C, Mirhaidari GJ et al (2020) Spontaneous reversal of stenosis in tissue-engineered vascular grafts. Science Trans Med 12(537):eaax6919 10.1126/scitranslmed.aax6919 PMC7478265 32238576

[CR34] Braeu F, Seitz A, Aydin R, Cyron C (2017) Homogenized constrained mixture models for anisotropic volumetric growth and remodeling. Biomech Model Mechanobiol 16:889–906 27921189 10.1007/s10237-016-0859-1

[CR35] Braeu FA, Aydin RC, Cyron CJ (2019) Anisotropic stiffness and tensional homeostasis induce a natural anisotropy of volumetric growth and remodeling in soft biological tissues. Biomech Model Mechanobiol 18(2):327–345 30413985 10.1007/s10237-018-1084-x

[CR36] Soleimani M, Muthyala N, Marino M, Wriggers P (2020) A novel stress-induced anisotropic growth model driven by nutrient diffusion: theory, fem implementation and applications in bio-mechanical problems. J Mech Phys Solids 144:104097

[CR37] Lamm L, Holthusen H, Brepols T, Jockenhövel S, Reese S (2022) A macroscopic approach for stress-driven anisotropic growth in bioengineered soft tissues. Biomech Model Mechanobiol 2:627–645 10.1007/s10237-021-01554-1 PMC8940864 35044525

[CR38] Holthusen H, Rothkranz C, Lamm L, Brepols T, Reese S (2023) Inelastic material formulations based on a co-rotated intermediate configuration application to bioengineered tissues. J Mech Phys Solids 172:105174

[CR39] Holthusen H, Brepols T, Linka K, Kuhl E (2025) Automated model discovery for tensional homeostasis: Constitutive machine learning in growth and remodeling. Comput Biol Med 186:109691 39842239 10.1016/j.compbiomed.2025.109691

[CR40] Szafron JM, Ramachandra AB, Breuer CK, Marsden AL, Humphrey JD (2019) Optimization of tissue-engineered vascular graft design using computational modeling. Tissue Eng Part C Methods 25:561–570 31218941 10.1089/ten.tec.2019.0086 PMC6791486

[CR41] Loerakker S, Argento C, Baaijens FP (2013) Effects of valve geometry and tissue anisotropy on the radial stretch and coaptation area of tissue-engineered heart valves. J Biomech 46:1792–1800 23786664 10.1016/j.jbiomech.2013.05.015

[CR42] Loerakker S, Ristori T, Baaijens FP (2016) A computational analysis of cell-mediated compaction and collagen remodeling in tissue-engineered heart valves. J Mech Behav Biomed Mater 58:173–187 26608336 10.1016/j.jmbbm.2015.10.001

[CR43] Sanders B, Loerakker S, Fioretta ES, Bax DJ, Driessen-Mol A, Hoerstrup SP, Baaijens FP (2016) Improved geometry of decellularized tissue engineered heart valves to prevent leaflet retraction. Ann Biomed Eng 44:1061–1071 26183964 10.1007/s10439-015-1386-4 PMC4826662

[CR44] Sesa M, Holthusen H, Lamm L, Böhm C, Brepols T, Jockenhövel S, Reese S (2023) Mechanical modeling of the maturation process for tissue-engineered implants: Application to biohybrid heart valves. Comput Biol Med 167:107623 37922603 10.1016/j.compbiomed.2023.107623

[CR45] Goriely A (2017) The Mathematics and Mechanics of Biological Growth, ser. Interdisciplinary Applied Mathematics, S. Antman, L. Greengard, and P. Holmes, Eds. New York: Springer Nature vol. 45

[CR46] Kuhl E, Steinmann P (2003) Mass and volume specific views on thermodynamics for open systems. Proc Royal Soc London Ser A Math Phys Eng Sci 459:2547–2568

[CR47] Coleman B, Noll W (1963) The thermodynamics of elastic materials with heat conduction and viscosity. Arch Ration Mech Anal 13:167–178

[CR48] Svendsen B (2001) On the modelling of anisotropic elastic and inelastic material behaviour at large deformation. Int J Solids Struct 38(52):9579–9599

[CR49] Reese S (2003) Meso-macro modelling of fibre-reinforced rubber-like composites exhibiting large elastoplastic deformation. Int J Solids Struct 40(4):951–980

[CR50] Gasser TC, Ogden RW, Holzapfel GA (2006) Hyperelastic modelling of arterial layers with distributed collagen fibre orientations. J R Soc Interface 3:15–35 16849214 10.1098/rsif.2005.0073 PMC1618483

[CR51] Humphrey JD, Dufresne ER, Schwartz MA (2014) Mechanotransduction and extracellular matrix homeostasis. Nat Rev Mol Cell Biol 15(12):802–812 25355505 10.1038/nrm3896 PMC4513363

[CR52] Humphrey JD, Cyron CJ (2022) Comment on tensional homeostasis at different length scales by d. stamenović and ml smith, soft matter, 2021, 17, 10274–10285. Soft Matter 18(3):675–679 34985470 10.1039/d1sm01151k

[CR53] Perzyna P (1971) Thermodynamic theory of viscoplasticity. Adv Appl Mech 11:313–354

[CR54] Fung YC (1990) Biomechanics: motion, flow, stress, and growth, 1st edn. Springer, New York

[CR55] Holzapfel GA, Gasser TC, Ogden RW (2000) A new constitutive framework for arterial wall mechanics and a comparative study of material models. J Elastic Phys Sci Solids 61:1–48

[CR56] Vladimirov IN, Pietryga MP, Reese S (2008) On the modelling of non-linear kinematic hardening at finite strains with application to springback comparison of time integration algorithms. Int J Numer Meth Eng 75(1):1–28

[CR57] Taylor RL (2020) “FEAP - finite element analysis program,” [Online]. Available: http://www.ce.berkeley/feap

[CR58] Korelc J (2002) Multi-language and multi-environment generation of nonlinear finite element codes. Eng Comput 18:312–327

[CR59] Korelc J (2009) Automation of primal and sensitivity analysis of transient coupled problems. Comput Mech 44:631–649

[CR60] Barfusz O, Brepols T, van der Velden T, Frischkorn J, Reese S (2021) A single gauss point continuum finite element formulation for gradient-extended damage at large deformations. Comput Methods Appl Mech Eng 373:113440

[CR61] Pacolli N, Awad A, Kehls J, Sauren B, Klinkel S, Reese S, Holthusen H (2025) An enhanced single gaussian point continuum finite element formulation using automatic differentiation. Finite Elem Anal Des 246:104329

[CR62] Eichinger JF, Paukner D, Szafron JM, Aydin RC, Humphrey JD, Cyron CJ (2020) Computer-controlled biaxial bioreactor for investigating cell-mediated homeostasis in tissue equivalents. J Biomech Eng 142(7):071011 32005993 10.1115/1.4046201 PMC7172870

[CR63] Ahrens J, Geveci B, Law C, Hansen C, Johnson C (2005) 36-paraview: An end-user tool for large-data visualization. The visualization handbook 717:1–50038

[CR64] Hunter JD (2007) Matplotlib: A 2d graphics environment. Comput Sci Eng 9(03):90–95

[CR65] Brandstaeter S, Fuchs SL, Biehler J, Aydin RC, Wall WA, Cyron CJ (2021) Global sensitivity analysis of a homogenized constrained mixture model of arterial growth and remodeling. J Elast 145(1):191–221

[CR66] Wirthl B, Brandstaeter S, Nitzler J, Schrefler BA, Wall WA (2023) Global sensitivity analysis based on gaussian-process metamodelling for complex biomechanical problems. Int J Num Method Biomed Eng 39(3):e3675 10.1002/cnm.3675 36546844

[CR67] Boehm CA, Donay C, Lubig A, Ruetten S, Sesa M, Fernández-Colino A, Reese S, Jockenhoevel S (2023) Bio-inspired fiber reinforcement for aortic valves: Scaffold production process and characterization. Bioengineering 10(9):1064 37760166 10.3390/bioengineering10091064 PMC10525898

[CR68] Figueroa CA, Baek S, Taylor CA, Humphrey JD (2009) A computational framework for fluid-solid-growth modeling in cardiovascular simulations. Comput Methods Appl Mech Eng 198(45–46):3583–3602 20160923 10.1016/j.cma.2008.09.013 PMC2770883

[CR69] Holthusen H, Lamm L, Brepols T, Reese S, Kuhl E (2024) Theory and implementation of inelastic constitutive artificial neural networks. Comput Methods Appl Mech Eng 428:117063

